# *In vitro* design of intrathecal drug administration therapies

**DOI:** 10.3389/fbioe.2025.1669537

**Published:** 2026-01-08

**Authors:** Ayankola O. Ayansiji, Caleb Gardner, Sebastien Dors, Daniel S. Gehrke, Francisco Moral-Pulido, Konstantin Slavin, Andreas A. Linninger

**Affiliations:** 1 Department of Bioengineering, University of Illinois Chicago, Chicago, IL, United States; 2 Department of Chemical Engineering, University of Illinois Chicago, Chicago, IL, United States; 3 UIC Student Intern From EPF, Ecole D’Ingénieur, Paris, France; 4 Department of Neurosurgery, University of Illinois Chicago, Chicago, IL, United States

**Keywords:** geometry-induced mixing, inversion of parabolic diffusion equation, intrathecal drug delivery, oscillatory cerebrospinal fluid flow, *in vitro* deformable spine model, drug infusion parameters, pharmacokinetic modeling

## Abstract

Due to the scarcity of reliable *in vivo* data, the pharmacokinetics of intrathecally (IT) administered drugs remain inadequately quantified. Designing new therapies is further hindered by variability in experimental methods, inter-individual and inter-species differences, and poor reproducibility across animal and human studies. To address these limitations, we developed an anatomically accurate, subject-specific replica of the cerebrospinal fluid (CSF)-filled spaces of the human central nervous system (CNS) using a multistep mold/casting process. The 3D-printed, transparent, deformable CNS phantom enables precise control of the infusion and physiological parameters, allowing systematic generation of reliable and repeatable biodispersion data for lumbar IT infusion protocols. Pulsatile artificial CSF flow within the closed system was tuned to replicate subject-specific stroke volumes and flow rates observed in MRI. The model’s optical clarity facilitated high-speed visualization and tracking of tracer dispersion, exceeding the temporal resolution of current neuroimaging techniques. An experimental series spanning physiologically relevant CSF and infusion conditions enabled quantification of the spatiotemporal distribution of IT-administered tracers. Inversion of the parabolic diffusion equation provided estimates of the coefficient of *effective dispersion*. A distributed pharmacokinetic model was used to evaluate the influence of chemical kinetics and mass transfer on tracer behavior. The proposed experimental apparatus for *in vitro* design of IT therapies offers a complementary or alternative approach to traditional trial-and-error animal studies.

## 1 Introduction

The blood–brain barrier presents a formidable obstacle to drug delivery (Niazi, 2023; Pandit et al., 2020). Intrathecal (IT) drug delivery is a minimally invasive modality that can overcome this limitation (Hettiarachchi et al., 2011; Lamer, 1994; Shoiche et al., 2007; Stearns et al., 2020). Two administration modes are common: (i) chronic dosing with an implanted drug pump and (ii) acute infusion through a lumbar catheter. Drug pumps operating with slow, pulsed, or steady infusion have a long successful application history for chronic pain and spasticity (Penn, 2003; Penn et al., 1989; De Andres et al., 2022; Hayek et al., 2021; Ghafoor et al., 2007). As far back as the 1980s, the IT drug system has been used for various indications, such as spinal stenosis, discogenic pain, and spasticity (Penn, 2003; De Andres et al., 2022; Hayek et al., 2021; Ghafoor et al., 2007; Bottros and Christo, 2014). Acute administration, usually with short intervals at relatively high injection rates (1 mL/min–3 mL/min), is a promising avenue for new therapies such as antisense oligonucleotides (ASOs) or enzyme replacement therapy targeting the brain (Cherry and Gourlay, 1987; Kwan, 1990; Ahmed et al., 2005; Wallace and Yaksh, 2000).

A major hurdle to effective administration of therapeutics to cerebral and spinal targets via IT delivery is the lack of data correlating diverse infusion protocols and cerebrospinal fluid (CSF) dynamics with the distribution speed and localization of the active drug molecule. Quantitative methods to predict the effect of the injection volume, infusate dose/dilution, and injection speed are incomplete. Trial-and-error experimentation in humans to ascertain the optimal infusion parameter is not feasible for several reasons. IT administration is invasive and associated with pain and infection risks even when carried out by expert clinicians (Saulino et al., 2016; Wesemann et al., 2014). It is almost impossible to carry out trials that systematically vary key parameters, including (i) the size and configuration of anatomical spaces, (ii) amplitude and frequency of CSF dynamics, or (iii) administering multiple drugs with different molecular properties. Ethically, it is challenging to justify exploratory trials in human subjects, yet parametric experimentation with repeats covering the extremes of physiological ranges is indispensable for robust statistical analysis.

To overcome these limitations due to data scarcity, we created an *in vitro* model of the human central nervous system (CNS) that replicates the main physiological and geometric characteristics of pulsatile CSF dynamics in humans. Our study focuses on quantifying physical transport of IT drug administration under different injection modes and across subject-specific physiological parameter ranges of CSF volume, pulsatile amplitude, and frequency.

Data from systematic trials with multiple repeats enabled the quantification of CSF-mediated drug transport as a function of injection modes and natural CSF parameters. We precisely characterized the *effective* bio-dispersion coefficient as a function of CSF amplitude and frequency. Based on the results, the tracer bio-dispersion matches the spatiotemporal dynamics of a *diffusive* dispersion process.

We conducted experiments with inert tracers to focus only on physical transport, avoiding interference from biological factors such as uptake or decay. Chemical kinetics for specific drugs was considered a separate step, which we addressed using pharmacokinetic (PK) modeling. Integration of drug transport data with biochemical reactions will be demonstrated through a computational study at the end of this article.

## 2 Methods

### 2.1 Manufacturing of the functional CNS model

An anatomically faithful, fully functional replica of the CNS was manufactured through a two-stage cast and mold process. We chose this technique over direct 3D printing because casting allows for a wider range of materials, including soft and transparent polymers that cannot be directly printed. Deformability is a critical requirement for creating natural CSF flow patterns inside closed anatomical spaces, which is impossible to achieve in open, rigid models. The five steps of the molding/casting process are presented briefly; a more detailed description is presented in Supplementary Appendix A. A flowchart of the manufacturing stages and processes can be found in Supplementary Appendix B.

#### 2.1.1 Imaging and segmentation

We acquired subject-specific anatomical properties of the human cranial and spinal SAS in several imaging sessions [see detailed MR protocol elsewhere (Tangen et al., 2015; Lu et al., 2016)]. The acquisition protocol of the human MRI data is presented in Supplementary Appendix A1; the original imaging data are provided in Ayansiji et al. (2023). DICOM image stacks of MR image data (Lu et al., 2016; Tangen et al., 2016; Tangen et al., 2015) were segmented using ITK-SNAP (Yushkevich et al., 2006; Associa and tion, 2003; Bidgood et al., 1997). The segmented data were structured into an inner and outer surface mesh encompassing the spinal SAS and stored in the stereolithographic (STL) format (with 53,514 elements) with good quality to retain anatomical details of the extracted images, especially the paired bundles of nerve roots. The image reconstruction is detailed in Supplementary Appendix A2.

#### 2.1.2 Mold design

**FIGURE 1:**
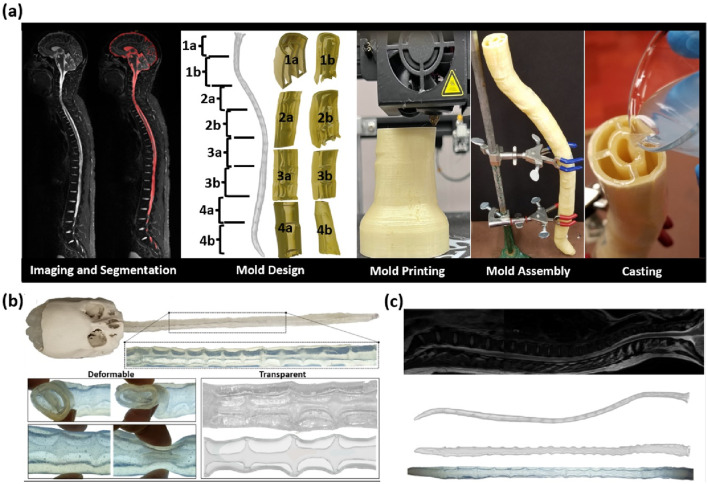
**(a)** Schematic diagram showing the five steps involved in the molding/casting process (i.e., imaging and segmentation, mold design, mold printing, mold assembly, and casting). **(b)** Casting result showing the deformability of the cast spine and transparency of the spine, allowing proper tracking of the tracer in the phantom during experiments. **(c)** Comparison of MRI data of the spine and the manufactured spine, demonstrating close reproduction of anatomical features. The final phantom dimensions closely match those obtained in MRI measurements, with average percentage differences of 1.88% in diameter and 3.78% in area.

We choose to design section molds in eight pieces to cover the entire neuraxis, as shown in Figure 1. Each piece was designed to measure less than 10 cm in length to fit into the regular commercial printing rig [Comgrow Creality Ender-5 3D Printer (Ender-5 3D Printer, 2024)]. Ring-like grooves were added to the connecting interfaces of each section using SolidWorks. The grooves/connections facilitated precise spacing and tight connections that are required in the subsequent assembly. The section meshes in the G-CODE format were loaded into a 3D printer [Comgrow Creality Ender-5 3D Printer (Ender-5 Plus 3D Printer, 2024)]. The total time required for printing all the segments was approximately 29 h. A detailed description of the mesh slicing process is provided in Supplementary Appendix A3.

#### 2.1.3 Mold printing

Each section, in G-CODE format, was 3D-printed using dissolvable polyvinyl alcohol (PVA) as filament in the mold printing stage (FDM technology). PVA properties are listed in Table 1, and a detailed explanation of the printing process, including the printer settings, is provided in Supplementary Appendix A4. The proposed sectioned design had the advantage of providing access to interior surfaces for smoothing and curating the rough edges of raw molds. The rough sections were smoothened with a brush and water, which ensured high surface quality of the mold, as shown in Supplementary Appendix Figure A2 in Supplementary Appendix A, and the final phantom.

**TABLE 1:** Characteristic properties of the PVA filament.

Property	Value/dimension
Roll diameter	3 (mm)
Filament roundness	+/− 0.07 (mm)
Filament diameter	+/− 0.05 (mm)
Net weight	0.5 (kg)
Recommended print temperature	160–190 (^0^C)
Solubility	Highly soluble in water

#### 2.1.4 Mold assembly

The eight section pieces were assembled and fastened using clear Elmer’s Glue (Elmer’s Products, Inc.) into a complete 3D mold of the spinal SAS (negative space). The alignment of the inner and outer mold pieces was precisely maintained by the printed spacer grooves to prevent mold breakage and ensure watertightness during casting, and the assembly was left to dry for 24 h (see Supplementary Appendix A5).

#### 2.1.5 Casting

TAP Platinum Silicone casting resin of shore A8 hardness (with Young’s modulus of 0.75 MPa) obtained from TAP Plastics (TAP Platinum Silicone, 2024) was poured in a way that 50 mL of the resin entered the closed mold in 1 min (i.e., approximately 50 mL/min flow rate), and then it was allowed to settle. TAP is transparent after casting and endowed with the final CNS analog (positive space) with soft, deformable, tissue-like properties. The TAP consists two sides, A (the base) and B (the catalyst), which are vigorously mixed and then poured into the assembled mold. The cast was left for 24 h to ensure full curing. This material was chosen because it produces an elastic wave speed of 27.2 m/s, which falls within the physiological range (3.5 m/s–33.8 m/s) (Kalata et al., 2009; Sonnabend et al., 2021; Sass et al., 2020). Further details on the casting process are provided in Supplementary Appendix A6. After curation, the entire mold/cast assembly was submerged in a water bath to gently detach and gradually dissolve the mold (negative) from the cast (positive space) by dissolving the water-soluble TPA. Figure 1c shows that the manufactured phantom replicates the geometry of the human spine, as indicated by the MRI data. A detailed description of the mold dissolution process is provided in Supplementary Appendix A7.

### 2.2 Functional dynamics reproduce natural CSF oscillations and enable tracer injection

#### 2.2.1 Pulsating flow

Pulsatile CSF motion in the human CNS is caused by oscillatory expansions of the cerebral blood compartment (possibly spread via the parenchyma) inside the rigid cranial compartment (Penn et al., 2011); the resulting volumetric strain is transmitted from cranial to spinal CSF, which in turn expands the spinal dural spaces. Distensibility is an essential feature of our closed deformable phantom, which cannot be achieved in open, rigid models. Accordingly, dural and pial boundaries of the CSF-filled spaces are deformable and closed, as shown in Figure 1b. To induce pulsatile CSF motion, a piston pump in the head compartment was used to expand and contract a water-filled balloon, mimicking a distensible blood/brain compartment. The balloon’s pulsations emulate the expansion of vasculature, which in turn sets in motion CSF flow from the cranial SAS into the elastically deformable spinal SAS.

**FIGURE 2:**
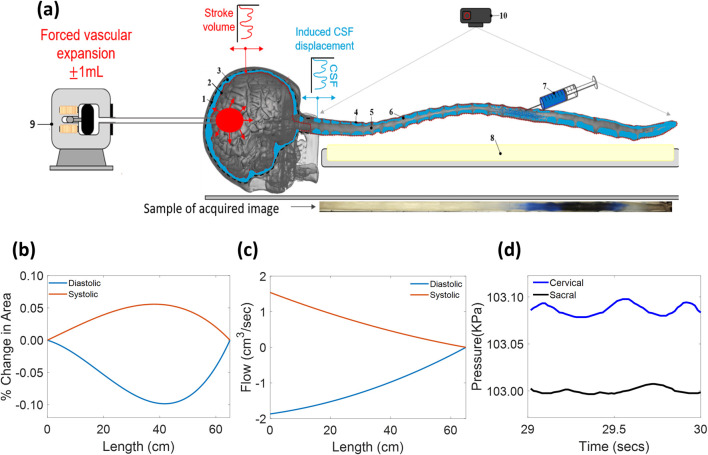
**(a)** Diagram of the CNS spine model in position on the benchtop for testing the infused tracer biodispersion. (1) Rigid cranial vault, (2) deformable brain parenchyma, (3) idealized cerebrospinal fluid, (4) deformable dura matter (subject-specific (S.s)), (5) spinal cord bundle (S.s), (6) peripheral nerve bundles (S.s), (7) infusion source, (8) illumination source, (9) piston pump (oscillations), and (10) optical recording camera. **(b)** Simulated area; **(c)** simulated flow. The flow is attenuated along the length of the neuraxis. **(d)** Measured pressure at different times. The temporal variation in the cross-sectional areas shows small canal deformations, which allows the pulsatile flow inside the distally closed CNS phantom. In addition, the closed model induces attenuated flow amplitude from the cervical region toward the sacral region, as shown in **(c)**, for diastole and systole.

*In vivo*, the volumetric expansion of the closed spinal SAS necessarily induces graded volumetric flow rates in the spinal CSF with snapshots in systole and diastole, as shown in Figure 2b. Because rigid models require open sacral and cervical sections, they enforce constant CSF flow along the neuraxis. The absence of deformability prevents graded flow amplitudes, resulting in unrealistic dispersion patterns. The proposed functionality enables us to cover the physiological range of stroke volume from 0.5 to 1 mL/beat (Hettiarachchi et al., 2011; Tangen et al., 2017). The dynamic area deformation and the pressure signal that drives the dispersion process are plotted in Figure 2c (obtained by simulation). The average change in area is 0.248 mm^2^, the average radial deformation is 1,383 μm, the average flow change is 0.78 mL/s, and the average change in pressure is 2.72 Pa. Pressure signals were measured *in vitro* using water manometrics, with the plots shown in Figure 2d.

#### 2.2.2 Tracer infusion system

For acute tracer infusion, we used a programmable syringe, as shown in Figure 2b to inject desired injection volumes at variable infusion rates. In the experiment, we injected 2 mL of tracer over a period of 1 min. Notably, our injection system is identical to clinical needle infusion, requiring no valves because the dura in the phantom is soft, watertight, and self-sealing.

#### 2.2.3 Optical tracking of the tracer fronts

After the 1-min injection, the syringe pump is stopped; further tracer motion occurs only due to natural CSF pulsation. Translucency of the dural surfaces allowed optical tracking with sufficient temporal resolution (120 fps) of intensity curves as a function of position along the neuraxis and time (see Figure 4a).

#### 2.2.4 Analysis of dispersion data

Video files showing tracer intensity plots were processed using video analysis software (Ivanchenko et al., 2010). MATLAB code that was built in-house was used for reconstructing the tracer intensity from snapshots at different times, as shown in Figure 4b. Our experiments did not allow accurate determination of radial intensity gradients, which appeared to be small. Therefore, radially averaged intensities (Ayansiji et al., 2023) were plotted. Because of asymmetry in biodispersion due to lumbar injection and the unequal size of the sacral and the cervical SAS, tracer profiles are not symmetric even in a perfect diffusion process. To account for skewed distributions, we designed a rigorous parameter estimation process termed *inversion of moments* as an improvement over the method of moments (MoM) (Ayansiji et al., 2023). We defined the *effective* biodispersion coefficient as the diffusivity of an ideal diffusion process that best matches the time course of the asymmetric second moment, the left-sided second moment, $M_{2,Left}\left(t\right)$ .

#### 2.2.5 Inversion of moments

Due to the asymmetry and boundedness of the spinal CSF spaces, second moment trajectories progressively taper off when the concentration profiles reach the sacral region. To overcome this departure from ideal diffusion (i.e., symmetric, infinite domain), we dynamically invert the time-dependent diffusion equation (Churchill, 1992) in Equation 2 to obtain an effective dispersion coefficient, *D*, which optimally matches the evolution of simulated moments to the non-linear trends observed in anatomical spaces. The inversion process can handle curved moment plots, thus providing more accurate estimates of the dispersion coefficient than MoM. We used moments in the objective of Equation 1 instead of raw concentration profiles to reduce uncertainties from noisy tracer-intensity measurements. Thus, the proposed moment inversion process (MIP) has integrative smoothing characteristics.

$$\min_{D}\epsilon \left(D\right)=\sum_{i=1}^{N}{\left(M_{2,Left}\left(t\right)-\phi \left(D,t\right)\right)}^{2}.$$ (1)

Here, $\epsilon \left(D\right)$ is the minimized error as a function of simulated diffusion, $\phi \left(D,t\right)$ is the left-sided second moment of the equivalent diffusion process for a given diffusion coefficient, *D*, at time point t, and N is the number of time periods considered. In order to obtain the desired optimum dispersion coefficient, *D* in Equation 1, the simulated moments $\phi \left(D,t\right)$ need to be computed by solving the parabolic diffusion (Equation 2), which is achieved numerically using the finite volume method (Linninger et al., 2023a), as shown in Supplementary Appendix C3.3. The optimum value that minimizes Equation 1 is considered the estimated *effective* dispersion coefficient. The non-linear inversion problem in Equation 1 was solved using MATLAB *fminunc*.

$$\frac{\partial C\left(x,t\right)}{\partial t}=D\;{\vec{\nabla }}^{2}C\left(x,t\right).$$ (2)

Here, C is the concentration, t is the time, and D is the *effective* dispersion coefficient. Experimentally observed left moments $M_{2,Left}\left(t\right)$ in Equation 1 are calculated using experimental image data using Equations 3 and 4.

$$M_{1}\left(t\right)=\frac{\int_{x_{o}}^{x_{m}}C\left(x,t\right)\;x\;dx}{\int_{x_{o}}^{x_{m}}C\left(x,t\right)\;dx}=\bar{x}\left(t\right),$$ (3)

$$M_{2,Left}\left(t\right)=\frac{\int_{x_{o}}^{\bar{x}\left(t\right)}{\left[x\left(t\right)-\bar{x}\left(t\right)\right]}^{2}\;C\left(x,t\right)\;dx}{\int_{x_{o}}^{\bar{x}\left(t\right)}C\left(x,t\right)\;dx}.$$ (4)

Here, x is the position along the neuraxis and $x_{0}$ and $x_{m}$ mark the lower and upper limits of the region of analysis, respectively. In each time frame, t, the first moment, $M_{1}\left(t\right)$ , provides the location of the center of gravity, $\bar{x}\left(t\right)$ ; $M_{2,Left}\left(t\right)$ is the left-sided mean spread of the visible concentration profile around its center, $\bar{x}\left(t\right)$ , which is obtained in the experiment. The results in Supplementary Appendix Table C1 show that inversion (MIP) was more accurate than the method of moments (MoM). This moment is determined by integrating over intensities to the left (in the cranial direction) of the mean position, $\bar{x}\left(t\right)$ , i.e., where $x\leq \bar{x}\left(t\right)$ . The least square error as a function of the dispersion coefficient, $\varepsilon \left(D\right)$ , between the observed left-sided second moment $M_{2,Left}\left(t\right)$ and the left-sided second moments of a simulated *equivalent* diffusion process ( $\phi_{i}$ ), was minimized. The value of diffusivity in the model that yields the least-squares difference between the observed and predicted moments is used as the objective in Equation 2. Equations 3 and 4 provide the first moment and the left-sided second moments of the concentration profile, respectively.

In addition, the first moment, $M_{1}\left(t\right)$ , of intensity plots along the neuraxis was computed as a function of time (total of 10 min at 1 min intervals) using Equation 3. Caudocranial motion and its speed [cm/min] were inferred from the slope of the $M_{1}\left(t\right)$ curve over time, which was obtained from the acquired intensity curves.

## 3 Results

### 3.1 Geometry validation of the mold and cast

To validate the geometric fidelity of the manufacturing process, Table 2 shows the cross-sectional geometry comparison between the MRI data, the printed mold, and the final phantom. Object dimensions were obtained using the measurement tool in 3D Builder and verified using a physical ruler. The dimensions of the final phantom closely match those obtained in MRI measurements, with average percentage differences of 1.88% in diameter and 3.78% in area. Figure 1b qualitatively shows that the phantom matched the anatomical features of the subject-specific MRI data. Gross geometrical dimensions of the dura, nerve roots, and S-like undulation of the CSF spaces along the neuraxis agreed with the anatomical features of the real human spine. The relatively small distortions were realized because of the flow rate control (slow pouring at 50 mL/min) of the casting material into the PVA mold.

**TABLE 2:** Validation of the manufactured spine dimensions at different locations (CM, cisterna magna; T2, thoracic; L1, lumbar; and S3, sacral).

Position	CM = 0 cm	T2 = 20 cm	L1 = 50 cm	S3 = 65 cm
*In vivo* diameter (cm)	1.30	0.70	1.00	0.78
Mold diameter (cm)	1.32	0.71	1.02	0.80
Phantom diameter (cm)	1.32	0.71	1.02	0.80
% difference in diameter^a^	1.54	1.43	2.00	2.56
Change in diameter (mm)^b^	0.01	0.02	0.005	0.003
*In vivo* area (cm^2^)	4.90	1.43	2.51	1.47
Mold area (cm^2^)	5.01	1.49	2.63	1.53
Phantom area (cm^2^)	5.01	1.49	2.63	1.53
% difference in area^c^	2.24	4.03	4.78	4.08
Change in area (mm^2^)^b^	0.20	0.49	0.10	0.05

^a^ Obtained by comparing the *in vivo* diameter with that of the manufactured phantom.

^b^ Obtained by simulation.

^c^ Obtained by comparing the *in vivo* surface area with that of the manufactured phantom.

#### 3.1.1 Dimensions of the human spine cross-sections

**FIGURE 3:**
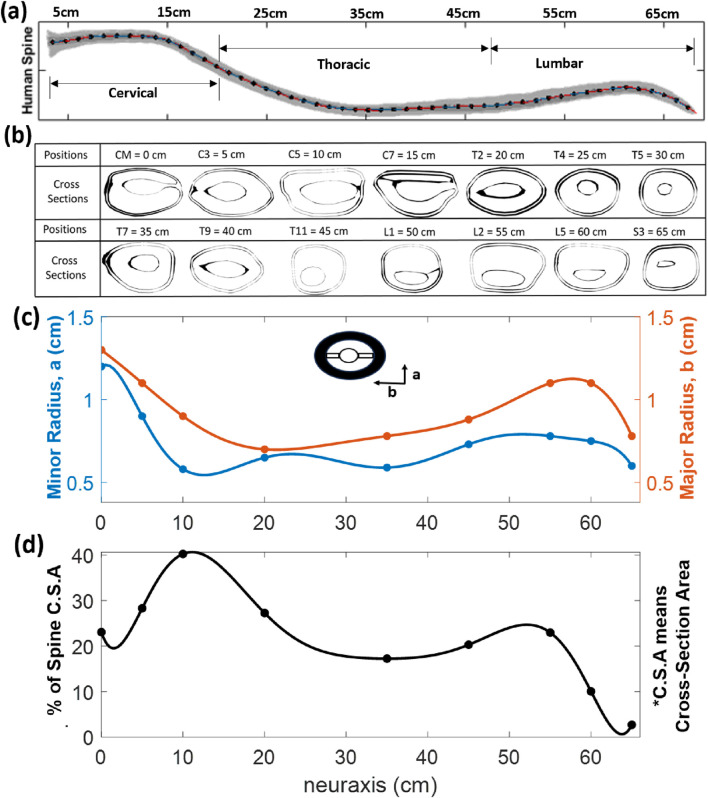
**(a)** Human spine with the regions. **(b)** Different cross-sections along the neuraxis at different positions. The ellipse shows the reference orientation for the major and minor radii. **(c)** Plots of the minor radius (dorsal to ventral extent) and major radius (right to left extent of the CSF space) distribution along the neuraxis. The cross-sections whose data were used for the interpolation are also placed at the corresponding points. The formula for the major and minor radii is listed in Supplementary Appendix E. **(d)** Percentage of the cross-sectional area covered by the spinal tissue. These data are needed to assess the CSF-covered area fraction in each cross-section.

The geometry of some selected cross-sections of the human spine is shown in Figure 3a, and their dimensions (minor and major radii) are presented in Supplementary Appendix Table D1 in Supplementary Appendix D. The sampled positions along the subarachnoid space are cisterna magna (CM), cervical region (C3, C5, and C7), thoracic region (T2, T4, T5, T7, T9, and T11), lumbar region (L1, L2, and L5), and the sacral region (S3), as shown in Figure 3d with their respective positions. The cross-sections are approximately elliptical, with b and a indicating the major and minor radii, respectively. Figure 3c shows the percentage of the computed cross-sectional area that is occupied by spinal tissue. Supplementary Appendix Table D1 in Supplementary Appendix D also shows the CSF-occupied area. More views of the cross-sectional areas are presented in Supplementary Appendix F.

### 3.2 Anatomically accurate deformable CSF model of the CNS

The closed deformable CNS analog replicates pulsating CSF flow in the spinal canal. Transparency of all system boundaries enables optical tracking of the tracer, as shown in Figure 2a. Cast specimens are durable (one phantom lasts approximately 100 experiment runs), adequately withstanding CSF flow oscillations without tearing or leakage. The average area deformation, pressure change, and flow change are 0.0021 cm^2^, 0.0204 mmHg, and 0.78 cm^3^/s, respectively (inferred by numerical simulation in terms of imposed stroke volume).

### 3.3 Systematic study of tracer dispersion in IT administration

#### 3.3.1 Effective dispersion

We optically tracked the tracer intensity in IT infusion experiments (N = 26) using trypan blue (from Millipore Sigma) using the setup in Figure 2a. A wide parameter range matching physiological and infusion modes was covered: the CSF frequency range was 40 bpm–127 bpm. The CSF cervical stroke volume was in the range of 0 mL/beat–1 mL/beat, and most experiments were run at 0.5 or 1.0 mL/stroke. All experiments (N = 26) were repeated three times; the repeats typically deviated from prior runs by less than 0.13 cm^2^/min.

Following the experimental setup shown in Figure 2a, 2 mL of the tracer (trypan blue) was injected into the spinal canal of the human spine phantom, which held water or artificial CSF (aCSF) at room temperature. IT injections lasted 1 min (phase 1), after which the syringe pump was stopped (phase 2). After stopping the injection, the tracer was allowed to disperse under the influence of the pulsation, as shown in Figure 2a. Both phase-1 and phase-2 occurred in the presence of natural CSF pulsation.

**FIGURE 4:**
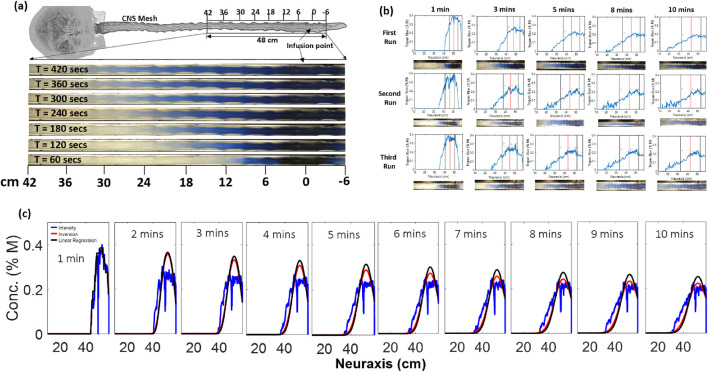
**(a)** Temporal evolution of the tracer dispersion (trypan blue) along the deformable model. Snapshots are taken each minute after the injection is already finished (t = 60 s). The x-axis label indicates positions along the neuraxis, and the point 0 cm marks the infusion point. Quotes of 42 cm (cervical) to −6 cm (sacral). **(b)** Typical raw concentration profiles obtained for different runs of the experiment at a frequency of 72 bpm and a stroke volume of 0.5 mL/stroke. **(c)** Plot showing the concentration profile predicted using the dispersion coefficient obtained via the inversion method (shown in red) and the method of moments (linear regression, shown in black). The blue curve shows the raw intensity curves acquired from the experiment for trypan blue at a frequency of 40 bpm and a stroke volume of 0.5 mL/stroke. Trends by inversion fit raw data better than those obtained by the method of moments.

A digital camera was used to record a video of the dispersion process (phase 2) over 10 min. In all our IT experiments, we observed that the tracer tends to move toward the cervical regions and the brain. The trypan blue intensity curves can be clearly seen expanding, initially almost symmetrically, from the lumbar injection site (x = 0) in the axial direction toward the sacral and thoracic regions. Once the concentration front hits the sacral boundary, dispersion profiles become skewed, and a caudocranial trend is clearly observed, as evidenced by a gradual shift of $M_{1}\left(t\right)$ from the injection site toward the cervical region. Snapshots taken at each minute, as obtained from the recorded video, are shown in Figure 4a. The intensity profiles of the acquired frames are noisy, so direct inference of the dispersion coefficient is not advisable.

The evolution of the first moment (the red vertical lines in Figure 4b) representing the center of gravity of the curve is indicative of the caudocranial motion of the tracer. The inversion process yielded the dispersion coefficients using the least-squares method (see Equation 1). Figure 4c shows the plot of the second moment for different runs, which shows that the second moment increases monotonically with time for the experiment at a frequency of 72 bpm and a stroke volume of 1.0 mL/stroke within the region of interest (ROI) of 10 cm–58 cm. The observed dispersion coefficients for the three runs using the inversion method are in the range of D = 6 cm^2^/min–8 cm^2^/min. Systematic parameter variation was performed across more than 70 experiments. The dispersion coefficients obtained (for trypan blue) using the method explained in Section 3.2 are shown in Supplementary Appendix G. Since the distribution of the tracer is solely based on the convective dispersion produced by the CSF dynamics, we expect the molecular diffusivity and thus the molecular weight to play a minimal role in dispersion. A theoretical basis for this assumption was provided by Moral-Pulido et al. (2023).

Figure 4c shows the comparison of intensities to predicted concentrations using MoM (Ayansiji et al., 2023), with results obtained through inversion (MIP). Although both methods exhibit similar overall trends, the concentration profiles using MIP values match the acquired raw intensity data more closely than those obtained using MoM.

**FIGURE 5:**
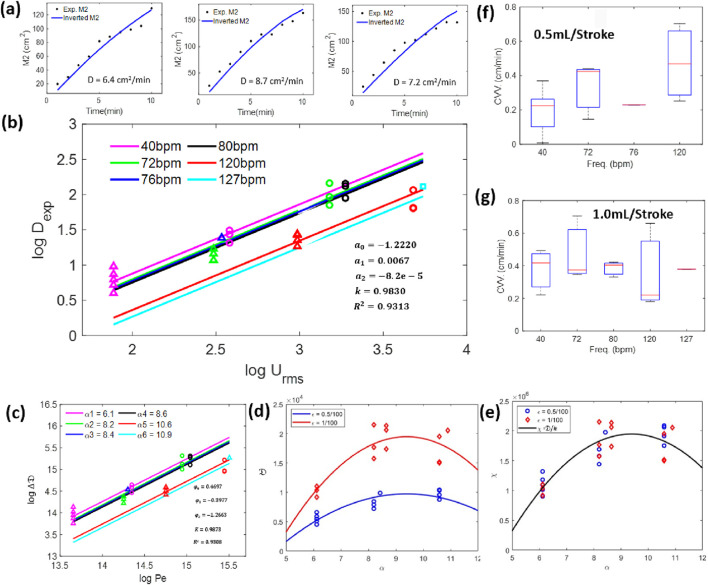
**(a)** Plots of second moments for the experiments and those from the inversion process obtained in three repeated runs. The inverted second moment follows the trend of those obtained from the experiment. **(b)** Relationship between the dispersion coefficient and frequency in the dimensional and **(c)** dimensionless forms for 26 experiments. **(d)** Dimensionless dispersion coefficient prediction curves as functions of the Womersley number (α) and the amplitude ratio (ε). **(e)** Normalized dimensionless dispersion coefficient prediction curve as a function of the Womersley number. **(f,g)** Caudocranial velocity (CCV) changes in the tracer relative to frequency for pulsation of 0.5 mL/beat **(f)** and 1.0 mL/beat **(g)**.

The results in Figure 5 summarize the dispersion coefficients as a function of CSF amplitude and frequency obtained in a set of experiments (N = 26). The data also show the reproducibility of the second moment trends using the inversion method.

Figure 5a shows three repeated runs of the experimental second central moments compared to the predicted moments obtained using the inferred (i.e., optimal) effective dispersion for the experiment conducted at a frequency of 72 bpm and a stroke volume of 1 mL per cycle. The simulated moments derived through inversion successfully capture the nonlinear, tapered trends observed in the data, which are attributed to anatomical asymmetries along the neuraxis affecting IT tracer dispersion. The temporal evolution of the experimental and predicted moments shows good agreement. Furthermore, all three experimental repeats under identical parameters (72 bpm and 1 mL stroke volume) produced consistent moment trends and inferred dispersion coefficients (D = 7.4 ± 1.9%). The similarity between the experimental moment profiles and the estimated coefficients (D_1_–D_3_) highlights the reproducibility of the MIP-based method despite unavoidable variability in lighting conditions across experiments.

##### 3.3.1.1 Functional dependence of the effective dispersion

The plots of Figure 5b show a clear correlation between the speed of dispersion and CSF stroke volume in raw dimensional form (amplitude of CSF pulsations expressed in terms of root-mean-square velocity, $U_{rms}$ , as defined in Equation H6 in Supplementary Appendix H). The dispersion coefficient, *D*, increases with CSF amplitude. Notably, repeat experiments show data points with the same abscissa, which provides a measure of the repeatability of the experimental procedure. Typical variance between repeat experiments was 2.13%. Parametric dependence of CSF frequency is also encoded as parallel lines.

The sequence of Figures 5c–e summarizes the relationship between the *effective* dispersion coefficient and CSF amplitude and frequency in dimensionless form. In the dimensionless form, two correlations were implemented, modeling the dispersion as a function of the non-dimensional numbers referring to the frequency and the amplitude. The first correlation uses suitable logarithmic scales, showing that the logarithm of the effective dispersion is linearly dependent on amplitude, with vertical shifts dependent on CSF frequency (ordinate offset). The second correlation uses scale analysis in the transport equation, which models the effective dispersion as a product of both the amplitude and frequency effects of CSF motion.

##### 3.3.1.2 Experimental dependence on root-mean-square velocity and frequency

A statistical correlation of the listed experimental dispersion coefficient in Supplementary Appendix G, D_exp_, as a function of natural CSF oscillations in terms of amplitude, root-mean-square velocity, $U_{rms}$ , and frequency, f , on a double logarithmic scale, is provided in Equation 5. A quadratic frequency dependence was also incorporated as an f-dependent offset, with $\lambda \left(f\right)=a_{0}+a_{1}\;f+a_{2\;}f^{2}$ , where $a_{0=}-1.2220,a_{1}=0.0067,\text{and }a_{2}=-8.2069\;x\;10^{-5}$ , and was drawn parametrically in Figure 5b. κ has a value of 0.9830 . Taking advantage of the logarithmic form of Equation 8, all parameters could be estimated simultaneously via linear regression, with the coefficient of determination for the best fit *R* ^2^ = 0.9313.

$$\log\left(D_{\exp}\right)=\lambda \left(f\right)+\kappa \;\log\left(U_{rms}\right).$$ (5)

##### 3.3.1.3 Experimental data in terms of dimensionless numbers (Womersley and Peclet)

Data could also be expressed in the dimensionless form, as shown in Equation 6. The dimensionless effective dispersion changes, $\Delta D=\frac{D_{\exp}-D_{0}}{D_{0}}$ , were plotted in Figure 5c based on the raw dimensional data of Figure 5b. The logarithmic correlations permit linear fitting for quantifying the dependence of dispersion change, $\log\left(\Delta D\right)$ , on the dimensionless oscillatory flow amplitude (Peclet number) and dimensionless frequency (Womersley number). Here, α is the Womersley number, $\alpha =\frac{D_{H}}{2}\sqrt{\frac{\omega }{\upsilon }}$ , $D_{H}=0.5cm$ is the hydraulic diameter, $\omega =\frac{2\pi f}{T}$ , f is the frequency in Hertz, T=60 secs is the period, $D_{0}={1.938\;x\;10}^{-6}$ cm^2^/min, and υ is the kinematic viscosity. The choices of dimensionless characteristics follow prior approaches used to describe transport in oscillatory flows (Hoagland and Prud’Homme, 1985).

$$\log\left(\Delta D\right)=\lambda \left(\alpha \right)+Κ\;\log\left(Pe\right).$$ (6)

The best fit parameter set ( Κ = 0.9873, $\left.\varphi_{0}=0.4697,\varphi_{1}=-0.3977,\text{and }\varphi_{2\;}=-1.2663\right)$ in Equation 6 has a coefficient of determination of *R* ^2^ = 0.9308, where $\lambda \left(\alpha \right)=\left(\varphi_{0}+\varphi_{1}\;\alpha +\varphi_{2\;}\alpha^{2}\right)$ . The dispersion coefficient of the tracers increases within the stroke volume, as shown in Figure 5c. In the case of frequency, it is noticeable that the increment is not monotonous; the dispersion coefficient stabilizes above a critical value of α , followed by a slight reduction of this parameter.

##### 3.3.1.4 Scale analysis

In addition to the statistical analysis of raw experimental data in the dimensional form in Equation 5, *scale analysis* of the advection–diffusion transport equation was performed to predict the *effective* dispersion coefficient (see details in Supplementary Appendix H). Two key parameters emerged from the non-dimensional transport equation: (i) the Womersley number, α , and (ii) the pulsatile amplitude ratio, $\varepsilon =\left(U_{rms}/\omega \right)/L$ . Scale analysis underscored the experimentally observed significance of amplitude and frequency but introduced the dimensionless volumetric deformation ratio, ε , thus pinpointing the value of the volumetric strain of the entire CSF-filled space as a characteristic number. Thus, the *effective* dispersion coefficient is also parameterized as a function of these two characteristics in Equation 7:

$$D=\varepsilon \;\lambda \left(\alpha \right),$$ (7)

where $D=D_{\exp}/D_{0}$ is the non-dimensional dispersion coefficient and $\lambda \left(\alpha \right)=\lambda_{0}+\lambda_{1}\alpha +\lambda_{2}\alpha^{2}$ is the Womersley-dependent function, with $\lambda_{0}=-5477\;x\;10^{3}$ , $\lambda_{1}=1580\;x\;10^{3}$ , and $\lambda_{2}=-84.09\;x\;10^{3}$ as best fits. This fit yields a coefficient of determination $R^{2}=0.9069$ , a match similar to that of the statistical model in Equation 6. Additionally, the dispersion coefficient can be rewritten in a *normalized* form, χ=D/ε , yielding a unique Womersley-dependent function. The dependence of the effective dispersion on both parameters is presented in Figures 5d,e: both panels show an increase in the dispersion coefficient within the Womersley number, reaching its maximum at α≃10 . However, the amplitude ratio scales the dispersion coefficient in an order-unity factor.

The comparison of the three models shows that the two main governing effects are related to the CSF pulsations, represented by its frequency (Womersley number, α ), and its amplitude, represented by its average velocity $U_{rms}$ (or Pe and ε ). The dependence of the *effective* dispersion as a function of both parameters is presented in Figures 5b–e: both approaches show that the amplitude of CSF motion scales the phenomenon, modulated by the flow frequency effect.

Each function presented in Equations 5–7 can be used to predict *effective dispersion* along the neuraxis as a function of subject-specific anatomical and CSF motion parameters (e.g., pulsatile frequency and amplitude). Quantification of the physical dispersion speed from this experimental series fills an important knowledge gap in the design of IT therapies. The integration of dispersion with biochemical kinetics is shown in Section 3.5.

#### 3.3.2 Speed of caudocranial motion

The caudocranial velocity (CCV) was also computed as the change in the first moment with time. Equations 7 and 8 show the correlation between the CCV and the frequency, f , and the root-mean-square velocity, $U_{\text{rms}}$ , respectively.

$${\text{CCV}}_{f}\;\left(\frac{\text{cm}}{\min}\right)=a\;f+b,$$ (8)

$${\text{CCV}}_{U_{\text{rms}}}\;\left(\frac{\text{cm}}{\min}\right)=a\;U_{\text{rms}}+b.$$ (9)

Here, a and b are 0.0018 and 0.2144, respectively, for Equation 8 and 0.0046 and 0.2650, respectively, for Equation 9.

Figures 5f,g show the relationship between the caudocranial velocity and oscillating frequency for both stroke volumes. As observed, there is a remarkable increase in the CCV, especially for the minimal stroke volume. However, increasing the CSF amplitude reduces the frequency-dependent effect, which becomes weak at higher frequencies.

### 3.4 Effect of nerve roots on tracer dispersion

**FIGURE 6:**
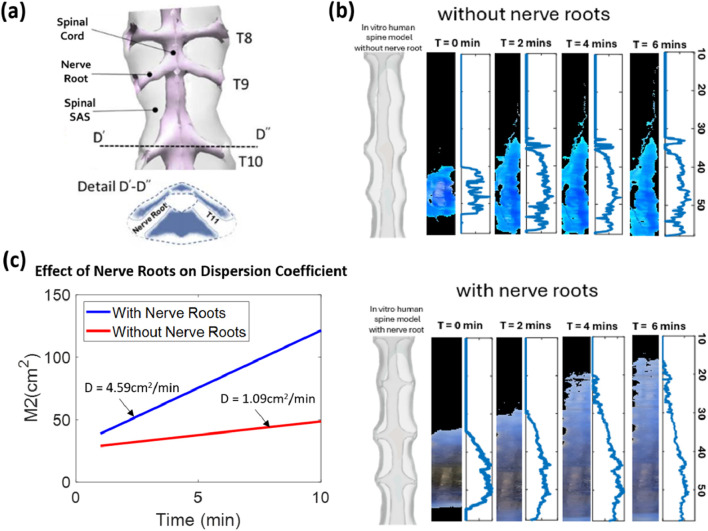
**(a)** Segment of the spinal SAS showing the detailed presence of the nerve root in the human spine. The line D′–D″ shows the detail of the nerve root in the spine. **(b)** Effect of nerve root on the dispersion of the tracer. The system with nerve roots has more than four times faster dispersion than that in which nerve roots were omitted in the manufacturing process. **(c)** Relation of the dispersion in a CNS spine model with peripheral nerve roots and without peripheral nerve roots.

We conducted experiments to quantify the effect of nerve roots on the speed of tracer dispersion. For this purpose, we manufactured a second CSN model in which dendrite ligaments and nerve root bundles were absent, as shown in Figure 6b (spinal canal without nerve roots), giving rise to a quasi-annular spinal SAS. Figure 6b shows the experimental results of the dispersion of tracer in the spinal canal with the inclusion or absence of micro-anatomical features. As shown in Figure 6b, the tracer spreads faster in the spinal model with nerve roots than in that without nerve roots. Biodistribution was 321.1% faster when nerve roots were present. The experimental results confirm our earlier findings (Hettiarachchi et al., 2011) and the predictions from direct numerical simulation (Hsu et al., 2012; Ayansiji et al., 2023; Linninger et al., 2023b; Tangen et al., 2020; Tangen et al., 2018), which reported that the nerve root enhances tracer dispersion in the human spine due to a phenomenon called *geometry induced mixing*. Figure 6c shows the quantitative representation of the effect of nerve roots. The 3–4-fold dispersion increase due to the presence of the nerve root accelerates drug dispersion under natural CSF oscillations. This benefit is active in high-volume infusion and under slow infusion via a drug pump.

### 3.5 Therapy design

**FIGURE 7:**
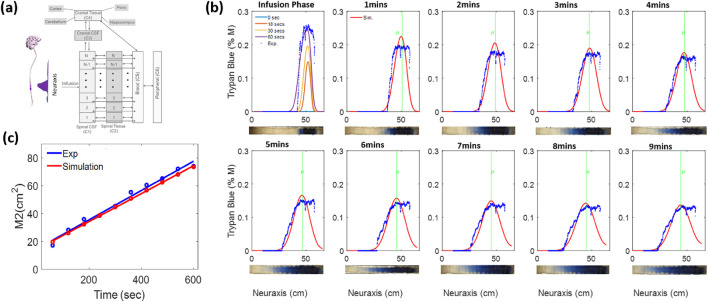
**(a)** Schematic diagram illustrating the derivation of the full pharmacokinetic model of drug dispersion. **(b)** Evolution of concentration profiles obtained using the pharmacokinetic model in Equation 10 without mass transfer and reaction. **(c)** Comparison of the second moment plots obtained from the experiment and simulation using the pharmacokinetic model in Equation 10.

How can physical dispersion data from this study be integrated with chemical kinetics and tissue uptake? We propose to predict drug biodistribution and uptake after IT administration using a distributed mechanistic PK model. A schematic diagram of the PK model with six compartments (C1–C6) introduced in Linninger et al. (2023a) is depicted in Figure 7a. It enables the simulation of injection and dispersion of active agents in the spinal CSF (typically lumbar injections) and tracking of drug concentration profiles in the spinal CSF, spinal tissue, cranial CSF, cranial tissue, blood, and peripheral, respectively (Linninger et al., 2023a). Notably, Equation 10 for the spinal CSF is an expanded version of the simple diffusion Equation 2, into which reaction and mass transfer terms have been incorporated.

$$\frac{\partial C_{1}\left(x,t\right)}{\partial t}=-\vec{\nabla }\left(u\left(x,t\right){\;C}_{1}\left(x,t\right)\right)+D_{eff}\;{\vec{\nabla }}^{2}C_{1}\left(x,t\right)-{\;k}_{1}{\;C}_{1}\left(x,t\right)-\dot{m_{1,2}}-\dot{m_{1,5}},$$ (10)

$${\dot{m_{1,2}}=\sigma }_{1,2}\left[C_{1}-K_{1,2}\;C_{4}\right],$$ (11)

$${\dot{m_{1,5}}=\sigma }_{1,5}\;\left[C_{1}-K_{1,5}\;C_{5}\right].$$ (12)

Here, ${\;C}_{1}\left(x,t\right)$ is the local concentration in the spinal CSF, $u\left(x,t\right)$ is the local velocity that comes from injection, $D_{eff}$ is the *effective* diffusivity of the tracer in the CSF, which is taken to be the same as D obtained experimentally via tracer distribution (cf Equation 2), ${\;k}_{1}$ is the first-order reaction kinetics in the spinal CSF, $\dot{m_{1,2}}$ is the rate of absorption in the spinal tissue, $\dot{m_{1,5}}$ is the rate of ASO leakage into the blood plasma, K is the distribution coefficient, and σ is the mass transfer coefficient.

The PK model integrates the respective influences of physical drug transport (dispersion), biochemical reactions, and mass transfer across CNS compartments (uptake). The left-hand side has drug accumulation (mol/min) expressed in terms of the drug concentration in the spinal CSF spaces, ${\;C}_{1}\;\left(x,t\right)$ . The convection flux is given by $-u\left(x,t\right)\nabla C1\left(x,t\right)$ , which accounts for the influences of high-volume injections on the CSF bulk flow velocity $u\left(x,t\right)$ and effects of deformations during high-volume injections. Drug dosing occurs via source terms at a specific location, x, and at instances in time according to the IT injection source $u\left(x,t\right)$ . It varies in time at different locations, x , along the neuraxis. CSF-mediated *physical transport* is modeled as effective diffusive flux and is given by $\left[D_{eff}\nabla C_{1}\left(x,t\right)\right]$ in terms of effective diffusivity, $D_{eff}$ , as determined in Section 4.3.3. *Chemical kinetics* accounts for the conversion of active drug species. The reaction terms can also be used to account for enzymatic inhibition or drug binding to proteins, which renders the active agent inactive or immobile. *Mass transfer* fluxes measure the amount of drug that is eliminated from the CSF and transferred into the spinal cord tissue (compartment 2), as shown in Figure 7a. The mass transfer from CSF to spinal tissue is provided in Equation 11. In addition, the drug can be taken up in the epidural space, which can be considered a drug loss for ASO therapy, as provided in Equation 12. This PK model can account for clearance and biological activities. The PK model was used to simulate tracer profiles in our IT infusion experiments with the reaction and mass transfer terms switched off, effectively replicating our bench tests *in silico*. Figure 7b shows a comparison between the profiles obtained experimentally and those predicted using the pharmacokinetic model. Simulated concentration profiles track the optical intensity plots; the second moment trajectories are in good agreement, as shown in Figure 7c.

Simulating the pharmacokinetics of an ASO lead molecule over several months of IT drug administration with multiple doses was achievable in just a few minutes using the proposed modeling framework (Linninger et al., 2023a). Supplementary Appendix Figure J1 shows results demonstrating the ability to implement multiple feeds and data on how drug binding affects the first and second moments. Unfortunately, detailed analysis of the interaction of transport and chemical kinetics is beyond the scope of this paper.

## 4 Discussion

**Human CNS phantom:** Using cast-and-mold techniques, we created a human spine model that is both anatomically and functionally accurate, including natural CSF pulsations with graded amplitudes. The phantom matches the real spine geometry as given by subject-specific MRI scans and reproduces tiny anatomical details such as the nerve roots. A key innovation of the CNS analog is that it is fully sealed and deformable, which is crucial for experiments aiming to replicate oscillatory CSF motion with an amplitude that is graded along the entire neuraxis of a dynamically deformable dural sac. Because the human spinal dura is flexible (Al-Habib et al., 2021; Beel et al., 1986), we also made the phantom’s dural boundary deformable using a casting material with appropriately configured mechanical properties. The casting material (TAP Platinum Silicone with a tensile strength of 218 psi) makes the phantom tear-resistant and durable, enabling it to withstand the stresses exerted by the pulsatile CSF motion, which is induced by the forceful piston action of the pump. The transparent casting material does not interact with or entrain microscopic tracer particles; its adsorption inertia makes it dependable for optical particle tracking. By carefully 3D-printing the cast and molding with an optimized catalyst mix ratio, the phantom achieves a precisely controlled thickness for the dura and excellent dimensional stability, with only 0.01% shrinkage. Controlling the thickness of the dural compartment in the manufactured phantom is essential because both its transparency and resistance to rupture depend directly on it.

**Applications:** The proposed CNS replica mimics *in vivo* CSF dynamics and physical biodistribution in the closed and deformable spinal subarachnoid space in humans. The design allows realistic implementation and quantitative performance testing of infusion protocols used in new drug trials (Linninger et al., 2023a) or in existing clinical practice for pain and spasticity management (Penn et al., 2011; Linninger et al., 2023b). Although this study focused on acute, high-volume lumbar injection experiments aimed at maximizing the caudo-cranial spread suitable for targeting the brain, the functional phantom can be deployed for bench trialing novel multi-dosing regimens with high-volume injections or chronic infusion protocols using drug pumps for individualized pain management. Thus, the human CNS phantom enables realistic performance testing and optimization of novel protocols, offering two key applications:

- *Systematic study of effective biodispersion.* By adjusting the CSF amplitude and frequency, we can systematically determine *effective dispersion* under realistic conditions of graded velocity profiles that are in effect globally across the neuraxis. This approach is a significant milestone toward validating the predictions of theoretical models for drug dispersion in oscillatory flows (Sánchez et al., 2018).
- *Design of new IT infusion protocols.* The transparent CNS surrogate allows for effective and inexpensive performance testing and optimization of new IT infusion protocols. The ability to manipulate the infusion and physiological parameters easily, along with high-speed optical tracking of tracers, may, in some cases, be able to replace animal trials, which do not scale to the human anatomy. The CNS phantom is suited for conducting pilot experiments to develop and optimize infusion protocols before clinical trials. Among different injection techniques, high-volume injections are well-suited for brain-targeted delivery because of the broader dispersion of the active pharmacological substrate. For a given injection time, high-volume infusions achieve larger dispersion distances than a drug pump, whose slow infusion rates would be relevant for spinal-located treatments.

**Experimental determination of *effective* dispersion:** The proposed bench setup enables adjustment of CSF flow dynamics—both amplitude and frequency—across a broad physiological range. Being able to freely vary CSF oscillations is critical for studying how changes in amplitude and frequency affect the dispersion speed, which in turn allows the description of physical transport using simple evidence-based formulas. The raw (dimensional) expressions for the dispersion coefficient effectively parameterize the speed of biodispersion, making it ideal for clinicians who wish to estimate how fast substances spread *in vivo*. Additionally, two alternative formulations—derived through scale analysis—were introduced to express the dependence of the observed tracer dispersion on dimensionless characteristics. The set of formulas can then be used to predict *effective dispersion* during human IT administration along the neuraxis with minimal computational cost.

**Image processing for tracer tracking tracer concentrations:** The proposed image analysis protocol overcomes unavoidable noise in tracer intensity curves due to uneven surface or lighting conditions and experimental variability. We previously showed the beneficial use of tracking second moments (Ayansiji et al., 2023) to suppress errors in raw intensity curves. The slope of the temporal evolution of the second moment (M2) directly yielded estimates of the effective dispersion coefficient, *D*. However, due to the asymmetry and boundedness of the confined spinal subarachnoid space, M2 trajectories depart from an ideal straight line as the tracer front progresses, thus potentially introducing a bias in slope determination, which, in effect, impairs the experimental determination of *D*. The proposed new analysis based on moment inversion (MIP) eliminated this shortcoming. MIP overcomes the deficiency of the MoM, helping to obtain better estimations of the true effective dispersion coefficient of the system. A comparison of the MIP and MoM results is given in Supplementary Appendix G showing a deviation of up to 56.2%.

**Pharmacokinetics:** We also integrated biochemical interactions and drug uptake with physical transport by developing a comprehensive PK model for drug dispersion following IT administration. The PK model briefly introduced for the central CSF spaces in Equation 10 enables the analysis of drug distribution across various biological compartments and allows computationally inexpensive simulation of diverse dosing scenarios—including multiple dosing, CSF production, and flushing with artificial CSF to accelerate cranial delivery. Notably, PK simulations of IT administration of ASOs over a period of several months were possible in less than 10 CPU minutes. The PK model analysis also confirms that for rapid delivery to the brain, flushing with CSF can be effective.

**Nerve roots:** We also confirmed experimentally that the effect of nerve roots is substantial. In a model without nerve roots, dispersion was four times slower than that in our main model with nerve roots. The experimental findings suggest that simplifying the assumption of smooth annular cross-sectional profiles on which several theories are based does not capture the significant geometry-induced mixing effects that occur only in the presence of micro-anatomical features. As a limiting point, our model does not contain dendrite ligaments and trabeculae, which are microfeatures present in the spinal anatomy whose effects were addressed in a previous study using *in silico* simulations (Tangen et al., 2015), showing that they give rise to a 2.5-fold increase in local flow velocities. We chose not to include trabeculae and dendrite ligaments in our phantoms because their number, orientation, and dimensions could not be resolved in our MR acquisitions.

Is IT dispersion a diffusive or convective process? The transport of solute in the human spinal canal has been studied by various authors (Linninger et al., 2023a; Sánchez et al., 2018; Lawrence et al., 2019; Hsu et al., 2012). Recently, several groups have proposed theoretical models of drug dispersion in smooth, idealized annular geometry without considering the spinal microanatomy (Lawrence et al., 2019; Sánchez et al., 2018). An elegant perturbation approach rendered time series results for species transport in the oscillatory fluid flow derived from the Navier–Stokes equations. The net effect of CSF oscillations on species transport was described as the combined effect of a Stokes drift and steady streaming introducing *effective convective terms* into the reduced-order species transport equations. The ability to match simulated concentration profiles obtained by direct numerical simulation (DNS) with results obtained with computationally much more efficient perturbation solutions (accounting for Stokes drift and steady streaming) significantly advances our theoretical understanding of species transport phenomena in oscillatory flow fields.

While the theory calls attention to *effective convection*, tracer dispersion observed in a complete CNS replica was consistently and adequately characterized by an *effective diffusion* process. We had previously stressed the significance of *geometry-induced mixing* around nerve roots based on a series of experiments that center on the formation of eddies that form around micro-anatomical features (Tangen et al., 2015; Ayansiji et al., 2023; Tangen et al., 2018). Our experimental evidence suggests that pulsating flow around obstacles causes local micro-mixing, which would have the globally observable effect of *diffusive (dispersive)* distribution of tracer molecules. It would be a significant development to characterize the global impact of microanatomy, such as ligaments, nerve roots, and trabeculae, in theoretical approaches (Bárcenas-Luque et al., 2024; Alaminos-Quesada et al., 2023). An important step in this direction is Parras-Martos et al. (2025), which was published just after the completion of this study.

An outstanding question remains whether geometry-induced mixing can be incorporated into more comprehensive theoretical descriptions. To facilitate the convergence of theory and CNS-wide experiments, we generated parametric data on cross-sectional dimensions along the spinal neuraxis to enable future comparisons, although these are beyond the scope of the current experimental study. The proposed elliptical shape approximations of the spinal anatomy might be useful for transferring data to theoretical models and validating simulation results against experimental evidence.

Our experiment also overcomes the limitations of rigid models that require artificial manipulation of CSF pulsations or lack the features needed to study the effect of realistic infusion settings. Until open theoretical questions are settled and the effects of infusion mode and spinal deformations are incorporated into existing theoretical models, experimental evidence in combination with the proposed pharmacokinetic modeling framework of this study could serve as a guide for IT therapy design.

## 5 Conclusion

A functional, anatomically accurate, fully sealed, and deformable model for the human CNS was manufactured by a mold-and-cast technique. The manufactured phantom reproduces natural CSF pulsations with graded velocity profiles over a wide range of physiological conditions by emulating the expansion of the blood vasculature in the closed cranial compartment. Tracers were successfully injected and tracked optically within the artificial CSF to quantify the influence of the infusion modes, drug dosage, and subject-specific anatomy and pulsations. To the best of our knowledge, our model is the first closed deformable bench experiment capable of reproducing CSF dynamics driven by cranial blood/brain expansion within the entire human CNS. The functional CNS analog might be useful for trials to optimize infusion protocols *in vitro* before human studies and for generating drug dosing guidelines for IT administration using commercially available pumps and catheters.

## Data Availability

The original contributions presented in the study are included in the article/Supplementary Material further inquiries can be directed to the corresponding author.
