# Supplementary material for: In vitro design of intrathecal drug administration therapies
Source: Front Bioeng Biotechnol. 2026 Jan 8;13:1669537. doi: 10.3389/fbioe.2025.1669537 (PMC12823945; doi:10.3389/fbioe.2025.1669537)
Supplement: Supplementary file 1 [file DataSheet1.pdf]

In vitro design of intrathecal drug administration therapies

Electronic Supplementary Information

Ayankola O. Ayansiji, Caleb Gardner, Sebastien Dors, Daniel S. Gehrke,
Francisco Moral-Pulido, Konstantin Slavin, Andreas A. Linninger

Appendix A. Detail explanation of the method involved in the manufacturing process.

A1. Acquisition of the Human MRI Data

MR images are an effective imaging technology for reconstruction of three dimensional detailed anatomical images. Subject specific phase contrast MR images were acquired, through a 29-year-old male volunteer, who signed the IRB approved consent form at the University of Illinois at Chicago. One of the MR images is shown in Fig. A1a. The 145 axial T2 weighted images of the spinal anatomy acquired have slice thickness of 5 mm, vortex size 0.547 x 0.547 x 5 mm, and matrix size of 256 x 256. The acquired image consists of the dura, spine, the nerve root, and CSF-filled space. Each of the images provides information on the dimension of the spinal anatomy, nerve roots position, volume of the spinal, cranial, and ventricular CSF spaces. MR images data was stored as DICOM – a nonproprietary data interchange protocol, digital image format, and file structure for biomedical images and the related information.

A2. Image Reconstruction and Mesh Generation

To reconstruct the geometry and mesh of the stored DICOM format images, ITK-SNAP was used for the segmentation process. The ITK-SNAP tool imports DICOM images and allows manipulation of each image/slice to highlight specific segments – in this case, the dura, and the nerve roots as shown in Fig. A1b. ITK-SNAP renders in the axial, coronal, and sagittal plane, during the highlighting process. This process allows segmentation from different planes, so segmentation is more precise. 3D reconstructed images are reconstructed in stereolithographic (STL) format. Post-processing of the reconstructed images, including filtering, filling of holes, and fitting the connectors, was performed with MeshLab, Mesh mixer and SolidWorks tools. The quality of the reconstructed image was maintained by applying only local smoothing to imperfections in the mesh as needed.

A3. Mesh Slicing for 3D Printing.

Printing the nearly 67 cm long spine is limited by the size of the 3D printing bed based on the size of the 3D printing machine used. To overcome this challenge, the whole spine mesh was divided into 8 pieces as shown in Fig. 1. Each of the pieces has both the spine (inner part) and the dura (outer part) with volume not more than 220 x 220 x 250 mm that can be printed effectively on the print bed. For easy reference each of the pieces was labelled with code as shown in Fig. 1. Slicing of the STL mesh is done using Cura 4.1.0 – an STL file slicing application which saves sliced segments into GCODE format, which is required coding language used by the Creality Ender 5 3D printer.

A4. 3D Printing of The Molds

The GCODE format mesh obtained was loaded into a 3D printer. A Comgrow Creality Ender-5 3D Printer was chosen as the ideal 'deposition' style filament printer since it has the functionality of a single extruder with a heated print bed. Also, the printer has print size of 220 x 220 x 300 mm, print precision of +/- 0.1mm, layer thickness of 0.1 – 0.4 mm, and nozzle diameter of 0.4mm. SainSmart dissolvable polyvinyl alcohol (PVA) filament was used for the printing, as shown in Fig. A1c. The PVA filament in the printer was pre-heated to a temperature of 190^0C to ensure

almost zero lagging in deposition of the PVA filament. The bed temperature was set to be 60^0C with PVA thickness of 0.15mm. Printing was done at full speed (180mm/s). PVA filament is used as mold because of its solubility in water, which makes it easy for the mold to be separated from the cast (the spine model). The printed mold usually has some roughness in the surface. To ensure high quality of the final phantom in terms of surface smoothness, the printed mold must be refined with the aid of water and brush to obtain mold with smooth surface as shown in Fig. A2.

A5. Assembling the Printed Pieces

The eight inner and outer component pieces of the phantom mold were assembled to form a whole phantom with water soluble, clear Elmer's Glue, as shown in Fig. A1d. The right portion (enough to make the pieces firm) of the glue was applied to avoid the alteration of the surface geometry of the phantom. Alignment of the inner and outer mold pieces relative to a central position is crucial to maintaining the correct geometry of the final cast silicone. To ensure alignment of the mold pieces in construction of the single mold 'unit' a roll of 20-gauge wire was used with lengths cut to bridge a gap between the inner and outer mold component pieces of each of the eight parts of the *phantom* assembly. The ends of these wire pieces are 'sunk' or pressed into the walls of the companion mold components. To ensure a watertight seal of the mold a ring of glue at the intersection of wire and PVA contact was carefully placed. The assembled phantom required 24 hours to air dry before pouring any casting material. Wrapping around the phantom with wax tape is also necessary, since wrapping prevents silicone leaks, air bubbles, and early mold decomposition from water.

A6. Casting

Once assembly of the *phantom* mold is finished, the next stage of manufacture is casting. TAP Platinum Silicone casting resin of shore A8 hardness, obtained from TAP Plastics, is used to make the super soft and flexible phantom. This material was chosen for a material elasticity in the phantom to replicate similar cerebrospinal fluid (CSF) and physiological material properties of the real human spine. The casting resin has two sides called side A, which is the base, and side B, which is the catalyst. The two sides are mixed in the ration of 1:1. In this study, 150 ml of each were mixed to for the casting reagent. The mixture was stirred gently for about 2 minutes before being poured in the printed mold, as shown in Fig. A1e. The silicone was poured in volume increments of 30.0 to 50.0 mL at once, slowly per interval of 1.0 minute to give time for trapped air bubbles to rise to the surface of the phantom mold. The cast was left for 24 hours to ensure that it cured fully.

A7. Dissolving the Mold

After 24 hours of casting, the dissolvable PVA mold was extracted gently from the cast with water, scissors, and spatula. First, the whole system was dipped in water overnight for twelve hours to begin the process of the PVA dissolving on the outside layers. After the PVA has started dissolving, the outer part of the mold was removed gently. It takes little more time for the inner parts of the dissolvable mold to be removed as there is a need to allow enough water to enter the inner part of the spine to have the inner PVA fully dissolved. After all the PVA had been fully

dissolved, a clear and deformable phantom of the in vitro human spine was recovered as shown in Fig. A1f.

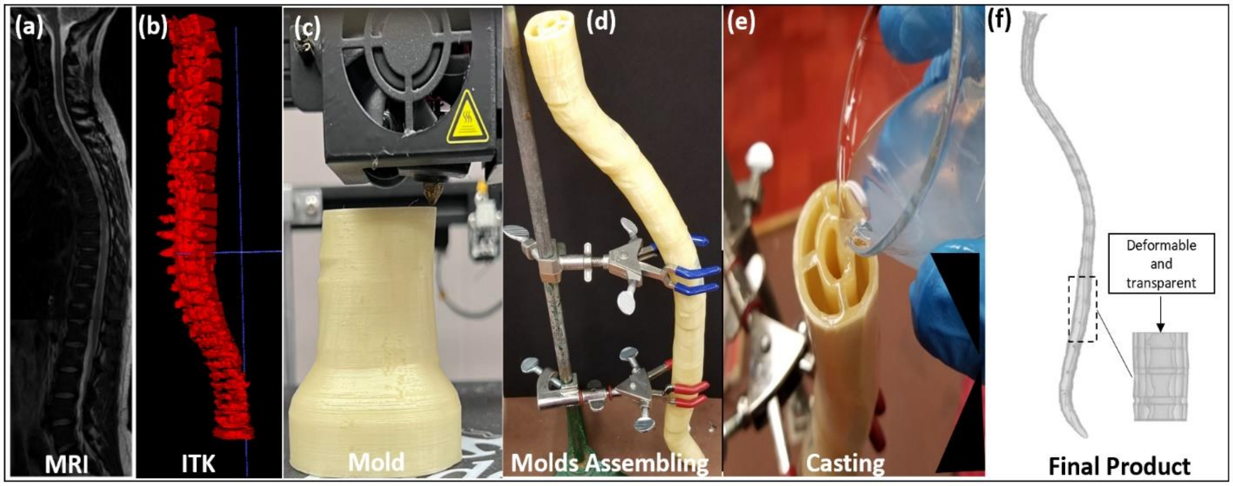

Fig. A1. The figure showing (a) The Human MRI data with the dura, spine, the nerve root, and CSF-filled space (b) The reconstructed image from the MRI data using ITK-SNAP tool (c) Mesh slicing for the 3D printing (d) 3D printing of a segment in the subject specific in vitro human spine model (e) Assembling of the 8 printed pieces (f) Casting resin which is the mixture of the base and catalyst (g) Fully immersed mold and cast in tank of warm water to dissolve the mold (h) The final product (deformable and transparent casted human spine)

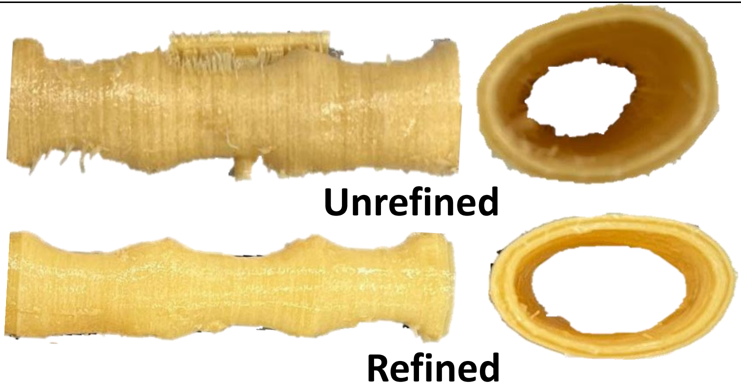

Fig. A2. The refinement of the printed phantom. The unrefined state consists of high roughness and some unwanted parts which will affect the quality of the final produced phantom if not treated.

Appendix B. The flow charts showing the 3D Printing, Casting, and Dissolving the Mold Process

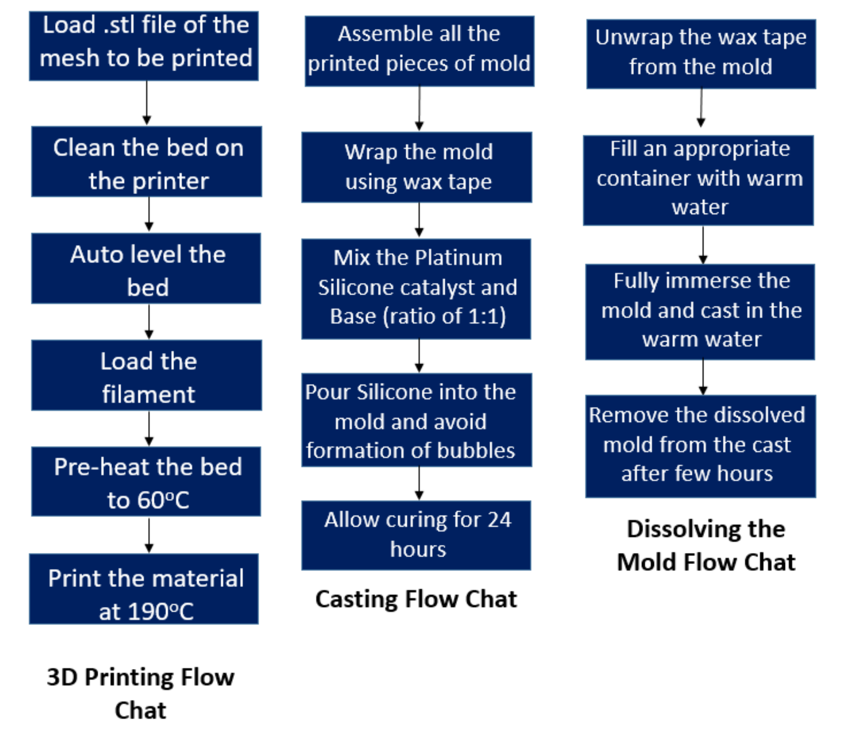

Fig. B1. The flow charts showing the 3D Printing, Casting, and Dissolving the Mold Process

Appendix C. Details on the inversion process for dispersion coefficients estimation

C1. First and Second Moments

The first and second moment can be computed using the concentration profiles as shown in Eq. (C1) and Eq. (C2) respectively.

$$M_1(t) = \frac{\int_{x_o}^{x_m} C(x,t)\, x\, dx}{\int_{x_o}^{x_m} C(x,t)\, dx} = \bar{x}(t) \quad \text{(C1)}$$

$$M_2(t) = \frac{\int_{x_o}^{x_m} [x(t) - \bar{x}(t)]^2\, C(x,t)\, dx}{\int_{x_o}^{x_m} C(x,t)\, dx} = \sigma(t)^2 \quad \text{(C2)}$$

Where x is the position along the neuroaxis, x_0 and x_m, marks the lower and upper limit of the region of analysis. In each time frame, t, the first moment, $M_1(t)$, gives the location of the center of gravity, $\bar{x}(t)$, The second moment can be interpreted as the mean spread of the visible concentration profile around its center, $\bar{x}(t)$. The increase in its variance with time, $\sigma(t)^2$.

Using the Eq. (C2), the second moment for all the computed profiles can be computed for the profiles obtained both analytical method or finite volume method. In the same way, using the Eq. (C1) and Eq. (C2), the first and second moment for the profile of the spread of the tracer obtained by experiment can be obtained. Different from what was done earlier where the second moment plots obtained from experiment was used to infer the dispersion coefficient of the experiment, here error minimization from inversion of dispersion process was done. Inversion of diffusion analysis to infer diffusion coefficient involves the simulation of diffusion process over a range of diffusion coefficient to obtain the diffusion coefficient that will give the second moment as that of the target profile. This method is usually done through error minimization between the simulated second moment and the target second moment.

To do the minimization analysis, the second moment plots obtained analytically from the concentration profile obtained using different diffusion coefficients and that obtained from the experiment were used to compute error as shown in Eq. (C3).

$$Error_i = \sum_{i=1}^{N} \left(M_{2_{sim}} - M_{2_{Target}}\right)^2 \quad \text{(C3)}$$

The diffusion coefficient whose error value is minimum was assumed to be the dispersion coefficient for the bio dispersion experiment. Where N is the number of diffusion coefficients simulated, $M_{2_{sim}}$ is the second moment obtained from simulation using either finite volume or analytical method concentrations profiles, $M_{2_{Target}}$ is the target second moment.

To obtain the diffusion coefficient that explains the process of the dispersion experiment, the plot of $Error_i$ versus the simulated range of diffusion coefficient is made. The minimum of the plot gives the inferred dispersion coefficient for the experiment.

C2. Left and Right Sided Variance

The process explained above involves the full analysis of the variance in the profiles. Another method was also studied which involves the analysis of the Left-sided and Right-Sided variance. This is to see how the symmetricity in the profiles affect the inferred dispersion process. This is necessary because the profile obtained in the experiment is not symmetric due to the point of infusion in the experiment which was at the lumbar region.

The left-sided second moment, $M_{2,Left}(t)$, consider the regions to the left of the mean position, $\bar{x}(t)$, i.e., where $x \leq \bar{x}(t)$. The left-sided second moment can be computed using Eq. (C4)

$$M_{2,Left}(t) = \frac{\int_{x_o}^{\bar{x}(t)} [x(t) - \bar{x}(t)]^2 \, C(x,t) \, dx}{\int_{x_o}^{\bar{x}(t)} C(x,t) \, dx} \tag{C4}$$

The right-sided second moment, $M_{2,Right}(t)$, consider the regions to the right of the mean position, $\bar{x}(t)$, i.e., where $x > \bar{x}(t)$. The right-sided second moment can be computed using Eq. (C5)

$$M_{2,Right}(t) = \frac{\int_{\bar{x}(t)}^{x_m} [x(t) - \bar{x}(t)]^2 \, C(x,t) \, dx}{\int_{\bar{x}(t)}^{x_m} C(x,t) \, dx} \tag{C5}$$

Following the above stated process, the error between the left and right-handed variance obtained in both the simulation and the one that was targeted are computed as shown in Eq. (C6) and (C7) for left-sided and right sided respectively.

$$Error_{i,Left} = \sum_{i=1}^{N} \left(M_{2_{sim,Left}} - M_{2_{Target,Left}} \right)^2 \tag{C6}$$

$$Error_{i,Right} = \sum_{i=1}^{N} \left(M_{2_{sim,Right}} - M_{2_{Target,Right}} \right)^2 \tag{C7}$$

Where $M_{2_{sim,Left}}$, is the left-sided second moment obtained by simulation, $M_{2_{sim,Right}}$, is the right-sided second moment obtained by simulation, $M_{2_{Target,Left}}$, is the left-sided second moment for the targeted profile and $M_{2_{Target,Right}}$, is the right-sided second moment for the targeted profile.

The diffusion coefficient that gives the inference of the dispersion coefficient for the experiment is obtained as explained above through minimization of error.

C3. Inversion of the Diffusion Analysis

C3.1. Symmetry System

As explained above, the inversion of the diffusion analysis helps to infer the dispersion coefficient of the experiments through method of error minimization. To do this analysis, first ground truth was laid by using a perfectly straight second moment plot ((Target M2 which was generated using the concentration profiles in Fig. C1a as obtained using injection at the middle of the neuraxis), with diffusion coefficient of 2.5cm^2/min) with no effect of the boundary layer (injection was made at the middle). Then concentration simulations were done using both finite

volume and analytical methods at a range of diffusion coefficients. Error minimization was done as explained above. In the case study used for this ground truth, the range of diffusion coefficients used is from 0.1 to 3 cm^2/min. The results of the recovered diffusion coefficient after the error minimization process are 2.5054cm^2/min and 2.4957cm^2/min for both the finite volume and analytical method respectively. To validate the estimated diffusion coefficient, the estimated coefficient was used for concentration profile simulation and the reproduced second moment plots were obtained. Fig. C1 shows the considered concentration profiles and the error plots. This analysis was done using both finite volume and analytical methods. The solution using the finite volume is presented in Fig. C1 below.

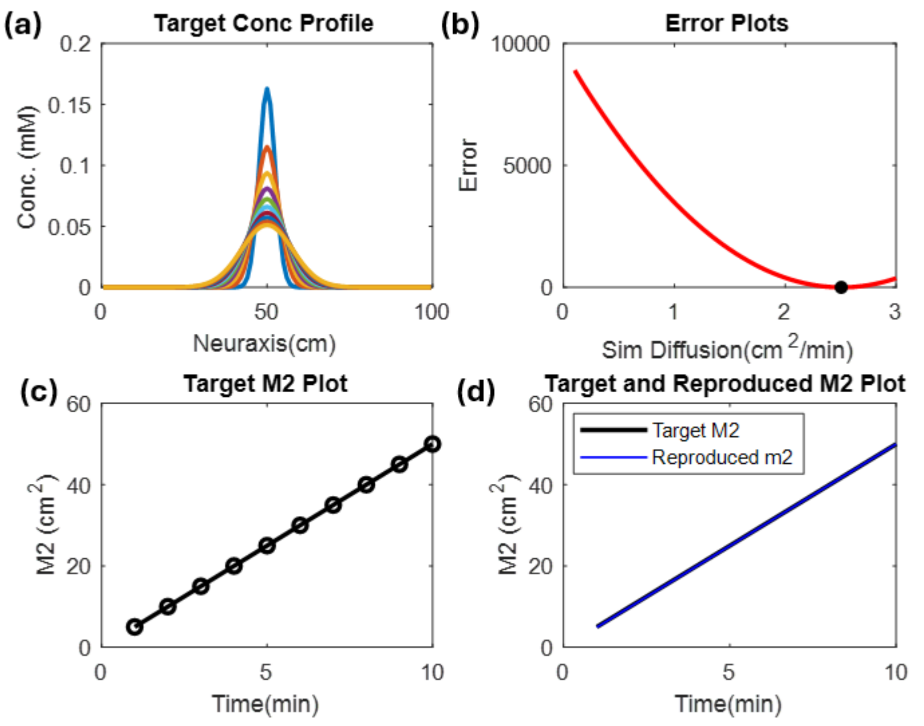

Fig. C1. (a) The concentration profile generated by the target diffusion coefficient where there is injection at the middle. (b) The minimized error obtained using Eq. (C3) at different simulated diffusion coefficients. (c) The plot of the second moment obtained from the target concentration profiles in Fig. C1(a). (d) The comparison of the plots of the target second moment with that reproduced using the diffusion coefficient obtained by the error minimization. This shows that the estimated diffusion coefficient is the same as the target diffusion coefficient. This analysis was done using the finite volume method.

C3.2. Asymmetry System

Another case study was done when there was lumbar injection. This is to investigate the effect of symmetricity on the inversion process since lumbar injection will make the profile not to be symmetric. The same process where different simulation of diffusion process using both finite volume and analytical methods was done as explained above to mimic concentration profile based on lumbar injection. Fig. C2 shows the results obtained for this analysis. The diffusion coefficient that gives the minimum error (when the average second moment was used) is 2.3308cm^2/min and 2.4957cm^2/min when finite volume and analytical methods were used respectively. These values are in very good agreement with the target or true diffusion coefficient of 2.5cm^2/min. Investigating Fig. C2d, the reproduced second moment in blue is in good agreement with the target second moment in black. The results in Fig. C2 are obtained using the finite volume method.

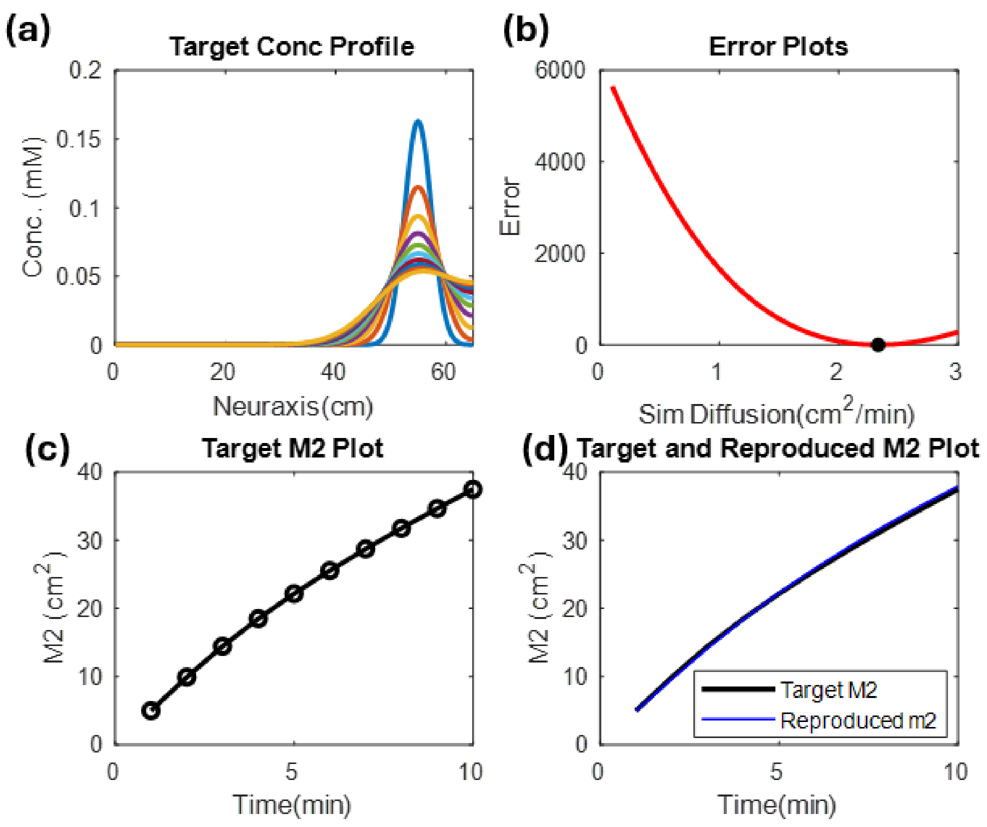

Fig. C2. (a) The concentration profile generated by the target diffusion coefficient where there is lumbar injection. (b) The minimized error obtained using Eq. (C3) at different simulated diffusion coefficients. (c) The plot of the second moment obtained from the target concentration profiles in Fig. C2(a). (d) The comparison of the plots of the target second moment with that reproduced using the diffusion coefficient obtained by the error minimization. This shows that the estimated diffusion coefficient is the same as the target diffusion coefficient. This analysis was done using the finite volume method.

Since there are different moments that can be considered in the case of asymmetry system, error minimization was done using the average second moment, left-sided second moment and right-sided second moment to investigate which moment gives the most accurate diffusion coefficient. Table C1 below shows the estimated diffusion coefficient with the use of different moments and different injection systems.

Table C1. The estimated diffusion coefficient obtained through inversion using different M2.

**Asymmetric (Lumbar) Injection System**			
Target Diffusion (cm^2/min)	Diffusion obtained using average M2 (cm^2/min)	Diffusion obtained using left-sided M2 (cm^2/min)	Diffusion obtained using right-sided M2 (cm^2/min)
Analytical Method			
2.5	2.4957	2.4957	2.4957
Finite Volume Method			
2.5	2.3308	2.3696	2.2823
**Asymmetric (Cervical) Injection System**			
Target Diffusion (cm^2/min)	Diffusion obtained using average M2 (cm^2/min)	Diffusion obtained using left-sided M2 (cm^2/min)	Diffusion obtained using right-sided M2 (cm^2/min)
Analytical Method			
2.5	2.4957	2.4957	2.4957
Finite Volume Method			
2.5	2.3308	2.2823	2.3696

Considering table C1, irrespective of the second moment that was used, the analytical method gives the same estimation of 2.4957 cm^2/min. However, when finite volume was used, the estimated diffusion coefficient varied based on the second moment that was used. Since the system of study involves lumbar injection, the inversion process using the left second moment gives the most accurate diffusion coefficient (2.3696 cm^2/min) among the second moments. The case is different when cervical injection system was used as the inversion process using the right-sided second moment gives the most accurate diffusion coefficient of (2.3696 cm^2/min). The full length of 0 to 65cm is used. Note: In the middle injection system, there is no difference in the three second moments as any of them can be used for the inversion process.

To have more understanding about the effects of all the moments, different combinations of the moments (first and second) were used as objective functions. Table C2 below shows the results of the diffusion coefficient estimated using the analytical and finite volume methods. Also, it must be noted that this is for the case of asymmetric (lumbar) injection and the target diffusion coefficient remains 2.5 cm^2/min.

Table C2. Table showing the estimated diffusion coefficients using different combinations of the moments as objective functions. (Asymmetric (Lumbar) Injection). Target Diffusion Coefficient is 2.5 (cm^2/min)

Combinations	M1 + M2(avg)	M1, M2(L), M2(R), M2(avg)	M1, M2(L), M2(R)	M2(L), M2(R), M2(avg)	M2(L), M2(R)
Analytical Method					
Estimated Diffusion coefficient (cm^2/min)	2.4957	2.4957	2.4957	2.4957	2.4957
Finite Volume Method					
Estimated Diffusion coefficient (cm^2/min)	2.3308	2.3405	2.3405	2.3405	2.3405

Where M1: First Moment, M2(avg): Average Second Moment, M2(R): Right-Sided Second Moment, M2(L): Left-Sided Second Moment

C3.3. Finite Volume Scheme

To carry out this inversion process explained above, the parabolic diffusion equation, Eq (C8) was solved using finite volume method.

$$\frac{\partial c}{\partial t} = D\, \vec{\nabla}^2 c \tag{C8}$$

The solution to Eq. (C8) using finite volume method can be obtained using Eq. (C9), in explicit form, and Eq. (C10) -Eq. (C12), in implicit form.

$$\frac{C_i^{m+1} - C_i^m}{\Delta t} = D\frac{C_{i+1}^m - 2C_i^m + C_{i-1}^m}{\Delta x^2} \tag{C9}$$

$$C_i^{m+1} = C_i^m + D\frac{\Delta t}{\Delta x^2}(C_{i+1}^m - 2C_i^m + C_{i-1}^m)$$

$$\frac{C_i^{m+1} - C_i^m}{\Delta t} = D\frac{C_{i+1}^{m+1} - 2C_i^{m+1} + C_{i-1}^{m+1}}{\Delta x^2} \tag{C10}$$

This leads to a system of linear equations for C_i^{m+1}.

$$C_i^m = -\frac{D\,\Delta t}{\Delta x^2}C_{i-1}^{m+1} + C_i^{m+1}\left(1 + 2\frac{D\,\Delta t}{\Delta x^2}\right) - \frac{D\,\Delta t}{\Delta x^2}C_{i+1}^{m+1} \tag{C11}$$

This can be written in matrix form as:

$$A\,C^{m+1} = C^m \tag{C12}$$

Where A is a tridiagonal matrix representing the co-efficient of C_{i-1}^{m+1}, C_i^{m+1}, and C_{i+1}^{m+1}, i is the control volume, m is the previous time step.

Appendix D. The table of dimensions of the radii and cross-sectional areas in the human spine

Table D1: Table showing the dimensions of the Human Spine Cross-Section.

Position	Neuraxis (cm)	a (cm)	b (cm)	Cross-Section Area (cm^2)	CSF Occupied Area (cm^2)
CM	0	1.2	1.30	4.90	3.77
C3	5	0.9	1.10	3.11	2.23
C5	10	0.58	0.90	1.64	0.98
C7	15	0.55	1.20	2.07	1.63
T2	20	0.65	0.70	1.43	1.04
T4	25	0.63	0.90	1.78	1.58
T5	30	0.53	0.73	1.22	1.13
T7	35	0.59	0.78	1.45	1.20
T9	40	0.68	1.00	2.14	1.57
T11	45	0.73	0.88	2.02	1.61
L1	50	0.80	1.00	2.51	2.08
L2	55	0.78	1.10	2.70	2.08
L5	60	0.75	1.10	2.59	2.33
S3	65	0.6	0.78	1.47	1.43

To get interpolated data in terms of cross section along the neuraxis. The equation that can be used to estimate the parameters a and b are shown in Eq. (E1) and Eq. (E2) respectively as shown by Lagrange interpolation in appendix F.

Note: The cross-section area is the total surface area of the slice and the CSF occupied area is the area without the area covered by the spine.

Appendix E. List of Lagrange interpolation formulars

Below are the developed equations for the Lagrange interpolations of minor radius, major radius, and the % of spine cross sectional area as shown in Eq. (E1), Eq. (E2) and Eq. (E3) respectively. For proper interpolation, some selected neuraxis points (0cm, 5cm, 10cm, 20cm, 35cm, 45cm, 55cm, 60cm, and 65cm) data were used.

$$a(x) = -\frac{9179 * x^8}{2027025000000000} + \frac{23057 * x^7}{18427500000000} - \frac{1145801 * x^6}{8108100000000} + \frac{33848567 * x^5}{4054050000000} - \frac{886491131 * x^4}{3243240000000} + \frac{1551687223 * x^3}{324324000000} - \frac{2894693 * x^2}{77220000} + \frac{1675367 * x}{45045000} + \frac{6}{5} \tag{E1}$$

$$b(x) = -\frac{503 * x^8}{116943750000000} + \frac{1657 * x^7}{1461796875000} - \frac{677 * x^6}{5568750000} + \frac{90713 * x^5}{13365000000} - \frac{5613031 * x^4}{26730000000} + \frac{354281 * x^3}{99000000} - \frac{41516 * x^2}{1299375} + \frac{3253 * x}{28875} + \frac{13}{10} \tag{E2}$$

$$s(x) = \frac{288949 * x^8}{1013512500000000} - \frac{15275389 * x^7}{202702500000000} + \frac{1483769 * x^6}{180984375000} - \frac{24881827 * x^5}{52650000000} + \frac{3563272663 * x^4}{231660000000} - \frac{12712410283 * x^3}{46332000000} + \frac{24858586679 * x^2}{10810800000} - \frac{104934369 * x}{20020000} + \frac{1153}{50} \tag{E3}$$

The unit of x is in cm.

Appendix F. More cross-sectional areas of the human spine.

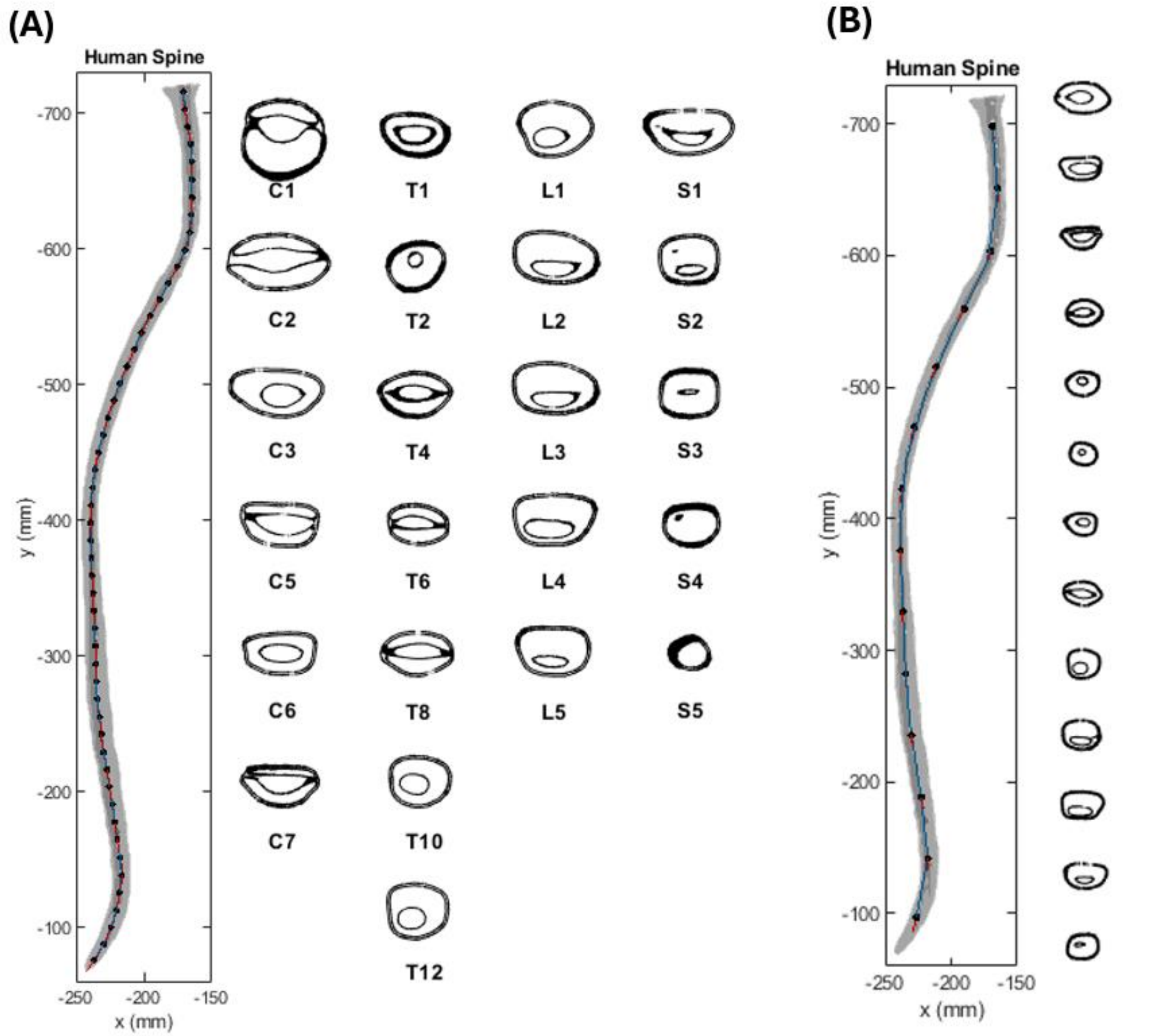

Fig. F. Figure showing the slices of the human spine obtained (A) at different regions of the spine (i.e., Cervical, Thoracic, Lumber, and Sacral regions). The labelling of the slices is placed under each of the slices. (B) at the interval of 50mm along the neuraxis.

Appendix G. The dispersion coefficients results obtained for the experiments for Trypan Blue

Table G1. Table showing the dispersion coefficients obtained through linear regression and inversion for Trypan Blue. Where Pe is the Peclet number, and α, is the Womersley number.

Freq (bpm)	Stroke volume (ml/stroke)	Run	D_{exp} (Regression) (cm2/min)	D_{exp} from Inversion (cm^2/min)	Pe (10^6)	α
40	0.5	1st	1.5291	2.2111	0.8514	6.12
		2nd	1.5036	2.0503		
		3rd	1.3162	2.6683		
		4th	1.0219	1.8241		
		5th	1.2406	2.3869		
	1	1st	2.9771	4.4472	10.7028	
		2nd	2.5899	3.7186		
		3rd	3.0256	4.1960		
72	0.5	1st	2.3600	2.9146	1.5480	8.20
		2nd	2.2049	3.1910		
		3rd	2.1993	3.4171		
	1	1st	4.2197	6.3668	30.960	
		2nd	4.5911	8.6935		
		3rd	4.1267	7.1558		
76	0.5	1st	2.5666	3.9950	1.6254	8.43
80	1	1st	5.4200	8.6432	3.4056	8.65
		2nd	5.4317	8.3417		
		3rd	4.8724	7.0352		
120	0.5	1st	3.1519	4.1704	2.5542	10.59
		2nd	2.7677	3.5427		
		3rd	2.9350	3.8693		
		4th	2.7170	4.2211		
	1	1st	4.3022	7.8894	51.084	
		2nd	3.8088	6.0804		
		3rd	3.9385	6.1307		
127	1	1st	4.6985	8.2915	54.180	10.90

Appendix H. Theoretical framework on determining the dispersion coefficient fitting using the scale analysis approach

Scale analysis – transport equation

The non-dimensional dispersion coefficient can be modeled by using the scale analysis technique onto the transport equation, to obtain the main governing parameters of this phenomenon.

This analysis starts with the general form of the transport equation. To that end, a variation of the governing equation described in the pharmacokinetic model of Linninger et al. [31] is represented in Eq (H1),

$$\frac{\partial C_1}{\partial t} + \nabla(uC_1) = D_{exp}\nabla^2 C_1 - kC_1 - \sum m_{1,i}, \tag{H1}$$

Where C_1 is the solute concentration, $u(x)$ represents the CSF flow velocity (pulsation-induced motion), D_{exp} is the dispersion coefficient of the solute, $k_1(x)$ is the ratio of drug absorption in the CSF (drug effectiveness), and $\sum m_{1,i}$ represents the total drug transfer in the other compartments (spinal tissue, blood, cranial cavity…). Since our experiments do not consider drug reaction nor mass transfer, Equation H1 is simplified to

$$\frac{\partial C_1}{\partial t} + \nabla(uC_1) = D_{exp}\nabla^2 C_1. \tag{H2}$$

We determine the governing dimensionless parameters of Eq (H2), based on CSF dynamics. The CSF motion in the spinal canal consists of a pulsatile motion synchronized with the cardiac flow with main frequency ω and average velocity U_{rms}. This fluid performs a back-and-forth motion in the spinal canal, represented as an annular, variable eccentric canal of total length L, hydraulic diameter D_H, and full filled of micro-anatomical features. Collecting the characteristic values of these parameters, together with the solute molecular diffusivity D_0 and the initial concentration C_0, Eq (H2) can be re-written by multiplying the non-dimensional values with their characteristic parameters, yielding

$$(\omega\, C_0)\frac{\partial C}{\partial t} + \left(\frac{U_{rms}\, C_0}{L}\right)\nabla(uC) = \frac{C_0\, D_0}{(D_H/2)^2}\,\mathfrak{D}\nabla^2 C. \tag{H3}$$

This equation can be divided by the angular frequency and the initial concentration, the resulting in

$$\frac{\partial C}{\partial t} + \varepsilon\nabla(uC) = \frac{\mathfrak{D}}{\alpha^2 S_c}\nabla^2 C. \tag{H4}$$

Here, Eq (H4) shows the dimensionless formulation of the transport equation, governed by the effect of the pulsatile amplitude through $\varepsilon = (U_{rms}/\omega)/L \simeq V_s/V$, the oscillating nature of the flow represented by the Womersley number, $\alpha = [(D_H/2)^2\, \omega/\nu]^{1/2}$, the molecular diffusion of the tracer, represented by the Schmidt number, $S_c = \nu/\kappa$, and the geometry-induced micro-mixing effect, represented by $\mathfrak{D}$. In our current S_c remains constant, so we reduce our analysis to the effects of the pulsatile amplitude and the oscillating nature of the flow. Thus, the non-dimensional dispersion coefficient can be defined as $\mathfrak{D} = \mathfrak{D}(\varepsilon, \alpha)$.

Fitting model

The previous analysis reduces the variability of the dispersion coefficient as a function of both flow parameters (amplitude and frequency). The flow nature correlates both parameters, modeling the dispersion coefficient as

$$\mathfrak{D}(\varepsilon, \alpha) = \psi(\varepsilon)\lambda(\alpha), \tag{H5}$$

Where $\psi(\varepsilon) = \varepsilon$ is the amplitude-related function, and $\lambda(\alpha) = \lambda_0 + \lambda_1\alpha + \lambda_2\alpha^2$ represents the frequency-related function. Under these considerations, the best fit is obtained with $\lambda_0 = -5477 \text{ x } 10^3$, $\lambda_1 = 1580 \text{ x } 10^3$, and $\lambda_2 = -84.09 \cdot 10^3$, yielding a substantial agreement with the experiments (determination coefficient $R^2 = 0.9069$). This model allows us to reduce the dispersion coefficient dependence by assuming the normalized function $\chi = \mathfrak{D}/\varepsilon$, being the Womersley number the unique independent parameter of this phenomenon.

Note:

$$U_{rms} = \sqrt{\frac{\int_0^t \left(\frac{V(t)}{A}\right)^2}{T} dt}\; V(t) = V_0 + \left(\frac{v_c}{2}\right) - \left(\frac{v_c}{2}\right)\cos(\omega t), \tag{H6}$$

$$A = \frac{V}{L}, \omega = 2\pi f$$

Where $V(t)$ is the instantaneous total CSF volumen, A is the hydraulic cross-sectional area of the spinal CSF SAS, T is the period, t is the time, V_0 is the initial volume of CSF in cm^3 in the system, v_c is cervical stroke volume, ω is the CSF angular pulse frequency, and f is the frequency in beats per minute.

Appendix I. The table of values for the pharmacokinetic simulation

Table I4. Table of values for pharmacokinetic study

Parameter	Value
Mass transfer from spinal CSF to spinal tissue space (U12)	0.01 mol/(sec.cm^2)
Mass transfer from spinal CSF to cranial CSF space (U13)	5.0 mol/(sec.cm^2)
Mass transfer from cranial CSF to cranial tissue space (U34)	5.0 mol/(sec.cm^2)
Mass transfer from cranial tissue space to blood (U45)	5.0 mol/(sec.cm^2)
Mass transfer from spinal tissue space to blood (U25)	0.1 mol/(sec.cm^2)
First order reaction constant (k1)	-1e-7/s
Diffusion coefficient (D)	0.05cm^2/s

Appendix J. Demonstration of multiple feeds injection and effect of drug binding on M1, M2 and AUC

Multiple feeds. Multiple injections of enzyme into the spinal CSF were simulated at different times between 0-60 secs (0.3 %M), the second injection was made between 600-660 secs (0.2%M), and the third injection was made between 900-960 secs (0.2%M). Fig. J1a shows the predictions of the pharmacokinetic model. There are spikes that coincide with the time of injections.

The table of value for the simulation is presented in appendix I. Fig. J1b shows the effect of reaction and mass transfer on the spread of the tracer in the spinal CSF, the trend of the first and second moment, and the behavior of the area under the curve. The rate of change of the first moment with time gives caudocranial velocity. The area under the curve (AUC) gives an idea of the conservation of mass. It was observed that reaction does not alter significantly the spread of the tracer (it however reduces concentration as there is up take by the spinal tissue). By integrating the chemistry, the AUC becomes smaller with time. As for the second moment, there is no change in the M2 when reaction was integrated. However, in the presence of mass transfer, M2 tends to become tapered with time.

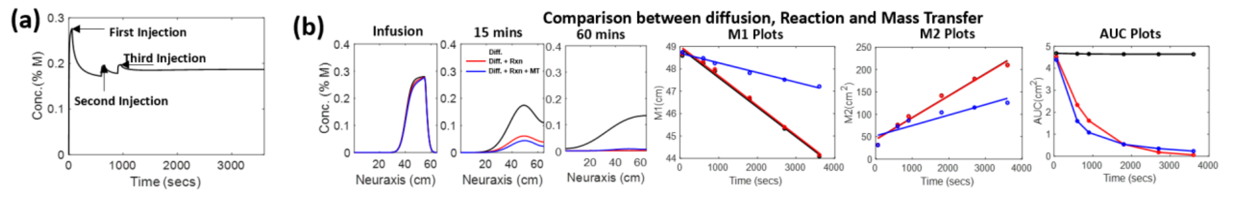

Figure J1. Figure showing (a) multiple injections made at different times using the pharmacokinetic model in Eq. (9). (b) comparison of the concentration profiles, M1, M2, and the (AUC) for the case of diffusion (black line), diffusion + reaction (red line), and diffusion + reaction + mass transfer (blue line). By integrating the chemistry, the AUC becomes smaller with time. As for the second moment, there is no change in the M2 when reaction was integrated. However, in the presence of mass transfer, M2 tends to become tapered with time.
